# Mental health first aid training for high school teachers: a cluster randomized trial

**DOI:** 10.1186/1471-244X-10-51

**Published:** 2010-06-24

**Authors:** Anthony F Jorm, Betty A Kitchener, Michael G Sawyer, Helen Scales, Stefan Cvetkovski

**Affiliations:** 1 Orygen Youth Health Research Centre, Centre for Youth Mental Health, University of Melbourne, Locked Bag 10, Parkville, Victoria, Australia; 2 Research and Evaluation Unit, Youth and Women's Health Service, Discipline of Paediatrics, University of Adelaide, South Australia, Australia; 3 South Australian Department of Education and Children's Services, Adelaide, South Australia, Australia

## Abstract

**Background:**

Mental disorders often have their first onset during adolescence. For this reason, high school teachers are in a good position to provide initial assistance to students who are developing mental health problems. To improve the skills of teachers in this area, a Mental Health First Aid training course was modified to be suitable for high school teachers and evaluated in a cluster randomized trial.

**Methods:**

The trial was carried out with teachers in South Australian high schools. Teachers at 7 schools received training and those at another 7 were wait-listed for future training. The effects of the training on teachers were evaluated using questionnaires pre- and post-training and at 6 months follow-up. The questionnaires assessed mental health knowledge, stigmatizing attitudes, confidence in providing help to others, help actually provided, school policy and procedures, and teacher mental health. The indirect effects on students were evaluated using questionnaires at pre-training and at follow-up which assessed any mental health help and information received from school staff, and also the mental health of the student.

**Results:**

The training increased teachers' knowledge, changed beliefs about treatment to be more like those of mental health professionals, reduced some aspects of stigma, and increased confidence in providing help to students and colleagues. There was an indirect effect on students, who reported receiving more mental health information from school staff. Most of the changes found were sustained 6 months after training. However, no effects were found on teachers' individual support towards students with mental health problems or on student mental health.

**Conclusions:**

Mental Health First Aid training has positive effects on teachers' mental health knowledge, attitudes, confidence and some aspects of their behaviour.

**Trial registration:**

ACTRN12608000561381

## Background

Mental health first aid has been defined as "the help provided to a person developing a mental health problem or in a mental health crisis. The first aid is given until appropriate professional help is received or the crisis resolves" [1]. To increase the mental health first aid skills of the general public, a Mental Health First Aid training course has been developed in Australia and has spread to many other countries [2]. This course teaches how to apply a mental health first aid action plan ("ALGEE") that involves the following actions: Assess the risk of suicide or harm; Listen non-judgementally; Give reassurance and information; Encourage appropriate professional help; Encourage self-help strategies.

A number of evaluation studies have been carried out on this course, including two randomized controlled trials, which have found improvements in mental health knowledge, reduction in stigmatizing attitudes, increased confidence in providing help and increased provision of help [3-10]. Mental Health First Aid training was initially developed to train adults to assist other adults. However, mental disorders often have first onset during adolescence and adolescents are particularly dependent on adults for recognition of the disorder, provision of appropriate support and referral to professional help [11]. To meet this need, a 14-hour Youth Mental Health First Aid course has been developed to teach adults how to assist adolescents with mental health problems [12]. Teachers may be well placed to take on this role, but have limited time available for in-service education. We therefore developed a modified and shortened version of the Youth Mental Health First Aid course to make it suitable for high school teachers and report here a randomized controlled effectiveness trial of this training.

## Methods

### Design

The study was a cluster randomized trial with schools as clusters and individual teachers the participants. A cluster design was used because it was not feasible to randomly assign individual teachers who were working in the same school because: (1) there may have been contamination of information provided across groups within the same school, and (2) schools may have responded to the training with changes in policy or procedures which would affect all teachers. Schools were randomly assigned to either receive training immediately or be placed on a wait list to receive training once the trial had finished. The trial has been registered with the Australian and New Zealand Clinical Trials Registry (ACTRN12608000561381).

### Participants

#### Individuals

Eligible participants were teachers of the middle years in school (i.e. Years 8-10, ages 12-15 years) at schools willing to participate in the study. Students taught by these teachers were also surveyed.

#### Clusters

Eligible clusters were all schools in the government, Catholic or independent systems in South Australia with Year 8-10 classes. These schools were sent a letter from the South Australian Department of Education and Children's Services explaining the study and inviting participation. Schools had to be willing to be randomized to do the training either in Terms 1 or 2 of 2008 (intervention schools) or Terms 3 or 4 of 2008 (wait-list control schools).

### Intervention

Teachers received a modified version of the Youth Mental Health First Aid course. To meet the scheduling needs of schools, the course was organized into two one-day parts of seven hours each. Part 1 was designed for all education staff and covered departmental policy on mental health issues, common mental disorders in adolescents (depressive and anxiety disorders, suicidal thoughts and behaviours, and non-suicidal self-injury) and how to apply the mental health action plan to help a student with such a problem. Part 2 was for teachers who had a particular responsibility for student welfare. It provided information about first aid approaches for crises that require a more comprehensive response and information about responses for less common mental health problems. Topics included how to give initial help to students who are experiencing a psychotic or eating disorder or substance misuse. Training was administered at the participants' school, with all available staff participating.

As documentation of the intervention, there was a lesson plan for each session, the existing Youth Mental Health First Aid manual [12] and a set of mental health factsheets. Lesson plans were developed by two Mental Health First Aid trainers of instructors who had previously worked as teachers. Additional material was added by staff of the Department of Education and Children's Services. Each course was conducted by two instructors, one from the Department of Education and Children's Services and the other from the Child and Adolescent Mental Health Service. These instructors received a one-week training program in how to conduct this modified Youth Mental Health First Aid course. They were trained by two experienced trainers, including Betty Kitchener who devised the Mental Health First Aid course.

### Objectives

For teachers, the hypotheses tested were that mental health first aid training improves the following: mental health knowledge, stigmatizing attitudes, confidence in helping students, helping behaviours towards their students, knowledge of school policies and procedures for dealing with student mental health problems, support given to colleagues with mental health problems, seeking information about mental health problems and their own mental health. The primary outcome measure for the trial was teacher knowledge.

For students, the hypotheses tested were that the mental health first aid training of their teachers would lead to an increase in the information they receive about mental health problems from their teachers, and that their mental health would improve.

All hypotheses pertained to the individual rather than the cluster level.

### Outcomes

The following teacher outcomes were measured at the individual level:

#### Knowledge about mental health problems

Teachers were administered 21 questions assessing information taught in both day 1 and day 2 of the course. Questions consisted of statements rated as "Agree", "Disagree" or "Unsure". The score was the number of questions answered correctly. Examples of items are: "Most adolescents with mental health problems get some sort of professional help", "It is not a good idea to ask someone if they are feeling suicidal in case you put the idea in their head" and "Depression can increase a young person's risk taking behaviour, e.g. reckless driving, risky sexual involvements".

#### Recognition of depression in a vignette

Teachers were given a vignette describing a 15-year old ('Jenny') with major depressive episode [13] and asked an open-ended question about what they thought was wrong with the person. Responses which mentioned "depression" were scored as correct.

#### Stigma towards depressed students

Teachers answered personal and perceived stigma items in relation to 'Jenny' [14]. Examples of personal stigma items are: "A problem like 'Jenny's' is a sign of personal weakness", "People with a problem like 'Jenny's' are dangerous", and "If I had a problem like 'Jenny's', I would not tell anyone". Perceived stigma items were the same except that they asked about what "most other people believe". These items were intended to be analyzed as scales based on a previous principal components analysis [14]. However, because the principal components could not be replicated in the teacher data, the responses to these questions were analyzed as individual items.

#### Beliefs about treatment of depression which are like those of health professionals

Teachers were given a list of 36 categories of people, medicines or other interventions and asked whether each of them is likely to be helpful, harmful or neither for 'Jenny'. Eleven of these interventions have been previously assessed by a consensus of clinicians as likely to be helpful [15]. The score was the number of these 11 interventions that teachers rated as likely to be helpful.

#### Confidence in providing help

Teachers were asked "How confident do you feel in helping a student with a mental health problem?" (Not at all, A little bit, Moderately, Quite a bit, Extremely). A parallel question was asked about confidence in providing help to a work colleague with a mental health problem.

#### Intentions to provide help to a depressed student

Teachers were asked "If you had regular contact with a student like 'Jenny', how likely are you to immediately: contact the family; discuss your concerns with another teacher; discuss your concerns with the counsellors; discuss your concerns with a member of the admin team; have a conversation with the student; talk to peers of the student; do nothing". Each item was rated on a 5-point scale from Never to Always.

#### Help provided to students

Teachers were asked in relation to the past month "Did you talk with a student about their mental health problem? (Never, Once, Occasionally, Frequently)". If yes, did you do any of the following: spent time listening to their problem, helped to calm them down, talked to them about suicidal thoughts, recommended they seek professional help, anything else".

#### First aid provided to colleagues

Parallel questions to those above were asked about first aid provided to colleagues, using the stem question "Did you talk with a school staff member about their mental health problem?"

#### School practices and policies

Teachers were asked in relation to the student in the vignette: "To what extent do you agree with the following as an important long-term strategy to support this student's learning and well-being: Review curriculum options/classroom practices; Review/change school policy; Set up planned family liaison; Set up planned community liaison; External support for student and family; Improve relationships within the school (i.e. teacher-student, student-student)" (Never, Rarely, Sometimes, Often, Always). Teachers were also asked the following questions in relation to the past month: "Did you discuss mental health problems of students with other teachers? Were mental health issues raised in staff meetings? Did you talk about your own mental health to a school staff member? Did you visit any websites giving information about mental health problems? Did you read any books or other written materials about mental health problems? (Never, Once, Occasionally, Frequently). Does your school have a written policy about how to deal with student mental health problems (Yes, No, Unsure)? Over the past month, how often did you put this policy into practice? (Never, Once, Occasionally, Frequently)."

#### Teacher psychological distress

Teachers completed the K6 Psychological Distress Scale [16].

The following student outcomes were measured at the individual level:

#### Recognition of depression in a vignette

Students were presented with the 'Jenny' vignette and asked the same recognition question that was used with teachers.

#### Stigma towards a depressed peer

Students were asked questions about personal and perceived stigma in relation to 'Jenny' [14].

#### Beliefs in the helpfulness of school staff for a depressed student

Student were given a list of 28 people or services, including a teacher and a school/student counsellor, and asked to rate them as likely to be helpful, harmful or neither for 'Jenny'.

#### Help received from school staff members

Students were asked "Over the past month, have you talked with a school staff member about any mental health problem you may have? (Never, Once, Occasionally, Frequently). If yes, did this person do any of the following: spent time listening to your problem, helped to calm you down, talked to you about suicidal thoughts, recommended you seek professional help, anything else".

#### Information received from teachers

Students were asked "Over the past month, have you received any information about mental health problems from your teachers? (Yes, No). If yes, how was this information presented: class lesson from teacher; poster, pamphlet, brochure or book; referral to website; talk from person other than the teacher; other".

#### Student mental health

Students completed the Strengths and Difficulties Questionnaire [17]. This is a 25 item questionnaire asking about how things have been for the young person over the last six months. The questionnaire yields subscale scores (5 items each) for emotional problems, conduct problems, hyperactivity/inattention, peer relationship problems and prosocial behaviour.

All outcomes were measured by printed questionnaires distributed by the school staff. Questionnaires to staff were administered at baseline (pre-test), immediately after training (post-test) and 6 months after (follow-up). Questionnaires were only provided to students whose parents gave consent. These questionnaires were administered at pre-test and follow-up only.

### Sample size estimation

Required sample size was estimated using software for power analysis in cluster randomized trials [18]. Likely effect sizes were taken from a randomized trial of Mental Health First Aid in a workplace setting [4]. In this workplace trial, recognition of the disorder in a vignette improved 10% in the intervention group compared to 1% in the wait-list control group. Similarly, advising someone to seek professional help increased by 10% vs 1%. To detect this effect in an unclustered trial with 80% power at the 0.05 significance level, required n = 200. The average school was estimated to have 30 teachers, giving a cluster size of 30. The intra-class correlation (ICC) was unknown. Examining ICC values from .01 to .10, the number of required clusters varied from 10 to 28. A previous cluster randomized trial of MHFA in a rural area [5] found ICCs ranging from 0.002 to 0.15, with most < 0.05. We therefore assumed an ICC of 0.05, which required a minimum of 18 schools to be randomized. We managed to recruit 16 schools for the trial, 14 of which participated as randomized.

### Randomization: sequence generation

The 16 schools were paired to be alike in socioeconomic characteristics. The pairing was carried out on the basis of: a scale of education disadvantage, size, location (metropolitan vs rural/remote), and gender (single vs mixed gender schools). Using the Random Integers option of Random.org, one school in each pair was randomly assigned to the immediate group and the other school to the wait-list group, by generating a 1 or a 2 for each pair (1 = immediate, 2 = wait-list).

### Randomization: allocation concealment

Allocation was based on clusters rather than individuals, so that all teachers at a school received the same intervention. Schools were told about the allocation before their teachers completed the pre-test questionnaire. This was necessary so that they could schedule the staff training days.

### Randomization: implementation

AFJ randomly assigned the schools. Participating schools were enrolled by a staff member of the Department of Education and Children's Services (HS) who informed them of their allocation after agreement to participate had been received.

### Blinding

Blinding of participants was not possible. Post-test and follow-up questionnaires were self-completed by teachers who knew whether they had completed the training or not. Students were not informed about whether teachers at the school had received training, but no systematic attempt was made to blind them.

### Statistical methods

The analysis of these multilevel or nested data required that the correlation of responses by individual participants between the measurement occasions and the correlation between participant responses within schools be taken into account. For that reason, mixed-effects models for continuous and dichotomous outcome variables, with group by measurement occasion interactions, were used to analyse the data. These maximum-likelihood based methods produce unbiased estimates when a proportion of the participants drop-out before the completion of the study, provided that they are missing at random [19,20].

In the current study, all the participants included in the analyses completed the first questionnaire. Twenty-two percent of teachers did not complete the post-test questionnaire and 28% the follow-up questionnaire. In relation to the students, 24% did not complete the follow-up questionnaire.

All analyses were performed using Stata Release 10 [21].

### Ethics

Ethical approval was given by the Youth and Women's Health Service Research Ethics Committee at the Women's and Children's Hospital.

## Results

### Participant flow

**Figure 1:**
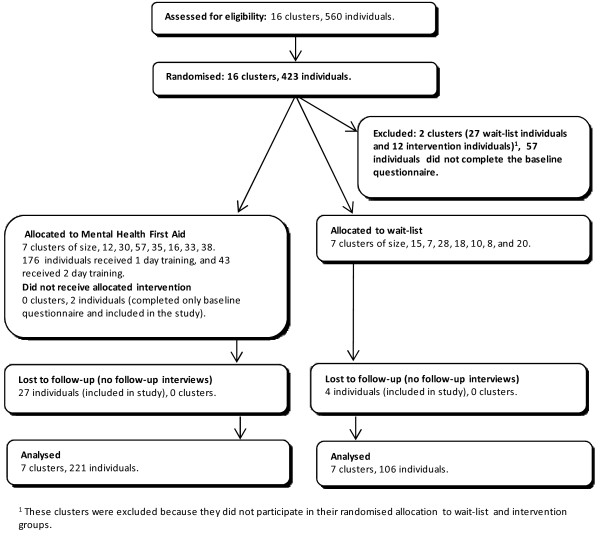
**CONSORT flow diagram**.

Figure 1 shows the flow of participants at each stage of the trial. Sixteen schools agreed to be randomized. Because the schools had to timetable their teacher training days early in the school year, the randomization had to be carried out before the baseline questionnaires were administered. After randomization and before baseline questionnaires, two schools decided that they were unable to follow the allocation because of changes in timetabling constraints. They would have to either withdraw from the study or else would agree to do the training in the period that was not allocated to them. In the interests of maximizing school participation, it was agreed to swap the allocation for these two schools (one from intervention to control and the other from control to intervention), resulting in 14, rather than 16 schools receiving the intervention as randomized.

### Numbers analysed

All participants who completed a pre-test questionnaire and were at one of the 14 schools that adhered to randomization were included in the analysis. However, a supplementary analysis was also carried out which included the 2 additional schools that did not adhere.

### Participants' Characteristics

Table 1 presents teacher and student demographic information. The teacher sample comprised 327 participants (221 in the intervention group and 106 in the control group), the majority of whom were female (65%). The most prevalent responses for the amount of teaching experience in schools were over 20 years (46%), and 3-5 and 6-10 years (13% respectively). In terms of the years of teaching at their current school, the most prevalent responses of teachers were 6-10 years (24%) and 3-5 years (22%). The main roles of the majority of teachers were classroom teacher (63%) and leadership (21%). The most prevalent subjects taught were English (28%), Studies of Society and Environment (27%), and Mathematics (22%). The student sample comprised 1,633 participants (982 in the intervention group and 651 in the control group), 54% of whom were female. Most students were aged 13 (38%) and 14 (33%) years, with the majority speaking English at home (92%).

**Table 1:** Teacher and student demographics

Characteristics	Intervention group	Control group	Total
**Teachers *n***	**221**	**106**	**327**
Gender *n *(%):			
Male	78 (35.3)	36 (34.0)	114 (34.9)
Female	143 (64.7)	70 (66.0)	213 (65.1)
Time working in schools *n *(%):			
Less than 3 years	24 (10.9)	4 (3.9)	28 (8.6)
3-5 years	30 (13.6)	13 (12.5)	43 (13.2)
6-10 years	28 (12.7)	13 (12.5)	41 (12.6)
11-15 years	22 (10)	7 (6.7)	29 (8.9)
16-20 years	22 (10)	14 (13.5)	36 (11.1)
More than 20 years	95 (43)	53 (51)	148 (45.5)
Time working in current school *n *(%):			
Less than 1 year	34 (15.4)	15 (14.4)	49 (15.1)
1-2 years	34 (15.4)	16 (15.4)	50 (15.4)
3-5 years	54 (24.4)	18 (17.3)	72 (22.2)
6-10 years	53 (24.0)	26 (25.0)	79 (24.3)
11-15 years	21 (9.5)	15 (14.4)	36 (11.1)
16-20 years	10 (4.5)	5 (4.8)	15 (4.6)
More than 20 years	15 (6.8)	9 (8.7)	24 (7.4)
Main role in school *n *(%):			
Leadership	38 (17.4)	28 (27.2)	66 (20.5)
Classroom teacher	146 (66.7)	58 (56.3)	204 (63.4)
Student welfare/counsellor	15 (6.9)	6 (5.8)	21 (6.5)
Support officer (SSO)	14 (6.4)	7 (6.8)	21 (6.5)
Other	6 (2.7)	4 (3.9)	10 (3.1)
Teaching subjects *n *(%):			
Arts	40 (18.1)	16 (15.1)	56 (17.1)
English	61 (27.6)	29 (27.4)	90 (27.5)
Technology	30 (13.6)	11 (10.4)	41 (12.5)
Language other than English	10 (4.5)	7 (6.6)	17 (5.2)
Studies of Society and Environment	57 (25.8)	32 (30.2)	89 (27.2)
Science	44 (19.9)	20 (18.9)	64 (19.6)
Physical Education	30 (13.6)	19 (17.9)	49 (15.0)
Mathematics	49 (22.2)	24 (22.6)	73 (22.3)
			
**Students *n***	**982**	**651**	**1,633**
Gender *n *(%):			
Male	451 (46.2)	295 (45.6)	746 (46.0)
Female	525 (53.8)	352 (54.4)	877 (54.0)
Age *n *(%):			
12	75 (7.7)	36 (5.6)	111 (6.9)
13	363 (37.4)	256 (39.9)	619 (38.4)
14	317 (32.7)	220 (34.3)	537 (33.3)
15	215 (22.2)	130 (20.3)	345 (21.4)
Grade *n *(%):			
7	31 (3.2)	8 (1.2)	39 (2.4)
8	403 (41.3)	293 (45.2)	696 (42.8)
9	308 (31.6)	208 (32.1)	516 (31.8)
10	234 (24.0)	140 (21.6)	374 (23.0)
Language spoken at home *n *(%):			
English	901 (92.2)	591 (91.2)	1,492 (91.8)
Another language	10 (1.0)	12 (1.9)	22 (1.4)
English and another language	66 (6.8)	45 (6.9)	111 (6.8)

With the exception of a significantly larger proportion of intervention group teachers having less than 3 years teaching experience in schools (10.9% vs. 3.9%, *χ*^2(1) ^= 4.42 , P = 0.036), and a smaller proportion in leadership roles (17.4% vs. 27.2%, *χ*^2(1) ^= 4.16 , P = 0.041), the characteristics of teachers were similar between the intervention and control groups. In relation to the student sample, the only significant difference in characteristics was that intervention group students had a significantly larger proportion of year 7 students relative to the control group (3.2% vs. 1.2%, *χ*^2(1) ^= 6.29 , P = 0.012).

### Teacher outcomes

Table 2 shows the descriptive statistics for teachers in the 7 intervention and 7 control schools, along with mean differences and odds ratios for pre vs. post and pre vs. follow-up intervention interactions, and their 95% confidence interval and significance level. More detailed analyses on these 14 schools, plus supplementary analyses including the 2 schools that did not adhere to randomization, are given in Additional File 1.

**Table 2:** Teacher outcome variables for intervention and control groups

	Intervention group			Control group			Mean diff./OR for pre vs post by intervention interaction (95% CI)	Mean diff./OR for pre vs follow-up by intervention interaction (95% CI)
				
	Pre	Post	Follow-up	Pre	Post	Follow-up		
**Mental Health Knowledge**								
Knowledge quiz: mean (SD)	11.14 (3.57)	13.07 (3.30)	12.68 (3.44)	11.26 (3.07)	11.11 (3.58)	10.76 (3.89)	2.08 (1.38-2.78)***	1.79 (1.06-2.52)***
Recognition of depression %	81.8	86.1	92.9	80.6	85.9	83.8	0.98 (0.27-3.56)	3.09 (0.77-12.43)
Beliefs about treatment for depression: mean (SD)	8.22 (2.39)	8.85 (2.54)	8.86 (2.39)	7.91 (2.44)	7.84 (2.74)	7.92 (2.46)	0.79 (0.23-1.34)**	0.73 (0.15-1.31)*
**Personal Stigma Items: % Strongly Disagree**								
Could snap out of it	32.1	40.1	37.3	31.1	29.6	26.4	2.12 (0.76-5.90)	2.59 (0.87-7.69)
Personal weakness	53.9	54.4	55.4	63.2	49.0	54.0	3.07 (1.16-8.14)*	2.47 (0.91-6.76)
Not real illness: %	45.0	47.1	48.7	43.4	37.8	34.5	1.70 (0.67-4.32)	2.50 (0.94-6.66)
People with that problem are dangerous	35.6	37.7	38.0	35.2	34.7	33.3	1.05 (0.39-2.82)	1.60 (0.57-4.45)
Best to avoid people with that problem	72.3	62.0	66.0	68.9	62.2	59.8	0.75 (0.30-1.89)	1.17 (0.45-3.03)
People with that problem are unpredictable	8.1	12.3	12.7	14.2	10.4	11.5	3.54 (0.88-14.17)	3.36 (0.82-13.83)
If they had problem they would not tell anyone	25.0	31.4	26.4	28.3	18.6	16.1	3.79 (1.34-10.71)*	3.42 (1.13-10.32)*
**Perceived Stigma Items: % ≥ Agree**								
Other people think could snap out of it	64.6	57.0	57.2	64.8	59.8	54.7	0.88 (0.34-2.26)	1.24 (0.47-3.33)
Other people believe a sign of personal weakness	52.7	52.9	56.0	58.5	56.7	45.9	1.10 (0.42-2.87)	3.01 (1.10-8.23)*
Other people believe not real illness	62.4	55.8	59.8	60.4	55.7	57.0	0.86 (0.37-2.02)	1.07 (0.44-2.60)
Other people believe they are dangerous	19.1	25.0	25.2	26.4	20.6	22.1	2.75 (0.98-7.66)	2.05 (0.72-5.85)
Other people would avoid people with that problem	23.6	29.7	28.9	27.4	23.7	24.4	2.42 (0.85-6.87)	1.90 (0.65-5.54)
Other people believe they are unpredictable	53.6	50.6	51.6	45.2	46.9	45.4	0.72 (0.31-1.68)	0.95 (0.40-2.28)
Other people would not tell anyone	61.4	59.1	51.6	67.6	51.6	52.9	2.57 (1.04-6.35)*	1.32 (0.52-3.36)
**Intended Helping Behaviours Towards Students**								
Contact the family: % ≥ *often*	38.2	41.8	44.0	36.2	37.5	35.3	1.28 (0.47-3.48)	1.46 (0.52-4.13)
Discuss with another teacher: % ≥ *often*	72.3	80.1	73.4	69.5	62.9	60.7	3.73 (1.31-10.62)*	2.46 (0.86-7.05)
Discuss with counsellors: % ≥ *often*	82.3	87.1	86.6	81.9	74.5	75.9	3.87 (1.21-12.41)*	2.98 (0.90-9.91)
Discuss with member of administration: % ≥ *often*	37.7	39.2	40.8	42.9	39.8	47.1	1.36 (0.52-3.60)	0.99 (0.37-2.68)
Have conversation with student: % ≥ *often*	68.6	72.5	70.3	61.0	58.2	49.4	2.06 (0.75-5.68)	3.16 (1.10-9.06)*
Talk with peers of student: % ≥ *often*	18.2	22.2	21.0	13.6	9.2	12.6	3.24 (0.91-11.54)	1.70 (0.49-5.94)
Do nothing: % *never*	65.5	66.1	66.5	69.5	65.0	61.6	1.95 (0.70-5.48)	2.37 (0.82-6.81)
**Help Given Towards Students: % ≥ Occasionally**								
Spoken with students about their mental health problems	52.1	52.1	54.8	53.3	51.0	47.7	1.34 (0.48-3.75)	1.73 (0.59-5.08)
Discussed a students' mental health problems with other teachers	67.9	72.4	66.2	70.5	68.4	58.1	1.87 (0.67-5.22)	1.91 (0.68-5.41)
Mental health issues raised in staff meetings	57.9	50.3	47.1	62.1	52.6	47.7	1.26 (0.51-3.07)	1.22 (0.48-3.08)
**Confidence in Helping Students and Staff with Mental Health Problems: % ≥ Quite a Bit**								
Confidence to talk with students about mental health problems	19.0	32.6	34.2	20.8	20.4	17.4	8.09 (1.89-34.63)**	7.02 (1.65-29.79)**
Confidence in helping a colleague with mental health problem	16.4	25.0	32.3	20.8	15.3	14.9	7.22 (1.84-28.4)**	11.65 (2.87-47.32)***
**School Policies on Student Mental Health**								
Review curriculum options/classroom practices: % ≥ *often*	54.3	56.7	58.0	59.1	48.5	41.9	2.22 (0.93-5.26)	3.76 (1.51-9.34)**
Review/changes school policy: % ≥ *often*	18.6	24.1	21.2	20.4	12.4	12.9	3.20 (1.12-9.14)*	2.44 (0.82-7.26)
Improve the relationships within the school: % ≥ *often*	65.6	69.4	68.2	71.4	61.2	58.1	3.09 (1.12-8.52)*	3.26 (1.14-9.27)*
School has written policy to deal with students with mental health problems: % *yes*	10.1	22.7	28.5	11.5	11.2	10.5	4.57 (1.28-16.26)*	7.28 (1.92-27.54)**
Policy been implemented in the last month: % ≥ *occasionally*	9.8	14.2	17.8	13.4	7.0	11.3	7.23 (0.85-61.37)	13.30 (1.32-133.44)*
**Interacting with Colleagues: % ≥ Occasionally**								
Talked with staff member about their mental health problem	39.1	38.0	38.3	38.4	38.1	36.1	0.88 (0.35-2.22)	0.93 (0.35-2.45)
Talk about own mental health problem with a staff member	35.8	39.4	38.2	37.1	34.7	34.5	1.49 (0.58-3.82)	1.23 (0.46-3.29)
**Seeking Additional Mental Health Information: % ≥ Occasionally**								
Visit any websites giving information about mental health	21.8	23.5	26.8	21.0	19.6	17.2	1.29 (0.42-3.91)	1.81 (0.56-5.79)
Read books or other written material bout mental health problems	43.9	49.1	39.9	38.1	38.8	35.6	1.30 (0.51-3.34)	0.85 (0.31-2.31)
**Teacher Mental Health**								
K6 6-24 (severe psychological distress) %	29.8	34.3	25.8	25.5	22.1	25.3	2.41 (0.77-7.49)	0.66 (0.20-2.13)
K6 3-24 (medium-high psychological distress) %	63.5	59.2	58.9	58.8	55.8	59.0	0.96 (0.34-2.70)	0.61 (0.20-1.85)

Legend: * p < 0.05; ** p < 0.01; *** p < 0.001

At post-test, teachers who received training had greater gains in knowledge (mean difference = 2.08, P < 0.001) and these gains were maintained at follow-up (mean difference = 1.79, P < 0.001). The teachers who did two days of training showed greater gains in knowledge than those who did only one day, but the difference was not significant. Recognition of depression was high at pre-test and was not affected by the training. Beliefs about the effectiveness of different approaches became more consistent with those of mental health professionals at post-test (mean difference = 0.79, P = 0.006) and this change was maintained at follow-up (mean difference = 0.73, P = 0.013). A number of personal stigma items showed improvement in response to training. Trained teachers were less likely than untrained ones to see depression as due to personal weakness (OR = 3.07, P = 0.024 at post-test and OR = 2.47, P = 0.077 at follow-up) and they were also less likely to be reluctant to disclose depression to others (OR = 3.79, P = 0.012 at post-test and OR = 3.42, P = 0.029 at follow-up). Two of the perceived stigma items showed changes, with the trained teachers more likely than the untrained teachers to believe that other people see depression as due to personal weakness (OR = 1.10, P = 0.848 at post-test and OR = 3.01, P = 0.031 at follow-up) and the trained teachers more likely to see other people as reluctant to disclose (OR = 2.57, P = 0.041 at post-test and OR = 1.32, P = 0.555 at follow-up). Intentions towards helping students showed some greater gains in the trained group, with trained teachers more likely to say that they would discuss their concerns with another teacher (OR = 3.73, P = 0.013 at post-test, OR = 2.46, P = 0.094 at follow-up), discuss their concerns with a counsellor (OR = 3.87, P = 0.023 at post-test, OR = 2.98, P = 0.075 at follow-up) and have a conversation with the student (OR = 2.06, P = 0.162 at post-test, OR = 3.16, P = 0.032 at follow-up). Confidence in helping a student with a mental health problem also increased (OR = 8.09, P = 0.005 at post-test, OR = 7.02, P = 0.008 at follow-up), as did confidence in helping a work colleague (OR = 7.22, P = 0.005 at both post-test and OR = 11.65, P = 0.001 at follow-up). Teachers who were trained were more likely to agree with the following strategies to support a student with a mental health problem: review curriculum options/classroom practices (OR = 2.22, P = 0.071 at post-test, OR = 3.76, P = 0.004 at follow-up), review/change school policy (OR = 3.20, P = 0.029 at post-test, OR = 2.44, P = 0.108 at follow-up), and improve relationships within the school (OR = 3.09, P = 0.029 at post-test, OR = 3.26, P = 0.027 at follow-up). Finally, trained teachers were more likely to report that the school had a written policy to deal with students with mental health problems (OR = 4.57, P = 0.019 at post-test, OR = 7.28, P = 0.003 at follow-up) and that the policy had been implemented in the previous month (OR = 7.23, P = 0.070 at post-test, OR = 13.30, P = 0.028 at follow-up).

Contrary to the hypotheses, training did not affect helping behaviours of teachers towards either students or colleagues, teacher mental health or seeking of information about mental health problems.

### Student outcomes

Table 3 shows the data on student outcomes from the 7 intervention and 7 control schools at pre-test and follow-up. More detailed analyses, plus supplementary analyses including the 2 schools that did not adhere to randomization, are given in Additional File 2. Very few student outcomes showed an impact of the training. The main one was that students of the trained teachers were more likely to report that they received information about mental health problems (OR = 2.60, P < 0.001), including a "class lesson from teacher" (OR = 2.76, P = 0.030), "poster, pamphlet, brochure or book" (OR = 4.84, P = 0.003) and "referral to website" (OR = 2.78, P = 0.045) (see Additional File 2). The only other change was in one item measuring stigma perceived in others, with increases in the perception that others believe in unpredictability (OR = 1.64, P = 0.006).

**Table 3:** Student outcome variables for teacher intervention and control groups

	Intervention group		Control group		Mean diff./OR for pre vs follow-up by intervention interaction (95% CI)
			
	Pre	Follow-up	Pre	Follow-up	
**Mental Health Knowledge**					
Recognition of depression *%*	56.4	68.1	58.5	70.5	1.03 (0.67-1.59)
**Beliefs and Intentions About Where to Seek Help for Depression**					
Help-seeking intentions - any adult source from 11 bullet point items^1^: *mean (SD)*	3.79 (2.76)	3.77 (2.91)	3.67 (2.61)	3.61 (2.81)	0.01 (-0.30-0.32)
Help-seeking intentions - all 11 adult source bullet point items above: *% yes*	2.2	2.8	2.2	3.0	0.90 (0.31-2.58)
Help-seeking intentions (all 5 items)^2^: *% yes*	9.3	10.1	7.2	8.2	0.91 (0.49-1.70)
Help-seeking beliefs (all 5 items)^3^: *% helpful*	23.9	24.0	20.4	20.5	0.96 (0.61-1.52)
**Personal Stigma: % Strongly Disagree**					
Could snap out of it	12.5	16.5	13.9	19.9	0.84 (0.51-1.40)
Personal weakness	12.3	14.6	15.5	19.5	0.89 (0.51-1.56)
Not real illness	15.4	17.6	17.8	20.7	0.96 (0.60-1.55)
People with that problem are dangerous	12.9	12.8	16.4	13.9	1.25 (0.76-2.06)
Best to avoid people with that problem	34.7	33.6	36.4	38.1	0.85 (0.58-1.25)
People with that problem are unpredictable	3.9	3.5	3.1	4.3	0.59 (0.25-1.41)
If they had problem they would not tell anyone	21.9	19.8	27.4	22.7	1.26 (0.81-1.96)
**Perceived Stigma: % ≥ Agree**					
Other people think could snap out of it	47.9	46.0	43.5	41.3	1.00 (0.71-1.42)
Other people believe a sign of personal weakness	52.2	53.0	52.5	46.9	1.42 (0.99-2.04)
Other people believe not real illness	43.1	41.4	46.2	38.6	1.33 (0.95-1.86)
Other people believe they are dangerous	37.4	38.2	39.0	34.4	1.34 (0.94-1.90)
Other people would avoid people with that problem	37.4	38.4	39.0	37.7	1.13 (0.79-1.61)
Other people believe they are unpredictable	44.1	47.6	53.7	48.2	1.64 (1.15-2.33)**
Other people would not tell anyone	48.0	47.6	48.4	46.0	1.07 (0.76-1.51)
**Help Received from Teacher**					
Talked with staff member about mental health problem: *% ≥ occasionally*	5.2	6.7	2.4	4.2	0.67 (0.28-1.62)
Received information about mental health problems: *% yes*	19.0	25.2	19.7	13.0	2.60 (1.68-4.05)***
**Student Mental Health**					
SDQ 20-40 (abnormal) %	9.1	9.6	7.0	10.3	0.51 (0.25-1.05)
SDQ 16-40 (borderline-abnormal) %	21.9	21.1	16.8	19.9	0.58 (0.33-1.01)
*SDQ Subscales*					
Emotional symptoms 7-10 (abnormal) %	9.4	9.2	8.1	8.5	0.84 (0.42-1.70)
Conduct problems 5-10 (abnormal) %	9.6	9.0	7.8	9.2	0.68 (0.35-1.32)
Hyperactivity 7-10 (abnormal) %	16.2	16.2	14.7	15.8	0.90 (0.52-1.57)
Peer problems 6-10 (abnormal) %	4.5	4.1	3.7	4.6	0.55 (0.21-1.45)
Prosocial behaviour 0-4 (abnormal) %	10.8	10.5	10.3	9.0	1.09 (0.59-2.02)

Legend: * p < 0.05; ** p < 0.01; *** p < 0.001

^1 ^The eleven intention items were nominating: a close family member, teacher, school/student counsellor, community member, pastoral care worker, community based religious leader, telephone helpline/counselling service, general practitioner or family doctor, child and adolescent mental health service, other mental health professionals (e.g., occupational therapist, social worker, nurse), and a youth health service.

^2 ^The five intention items included nominating: a school/student counsellor, telephone helpline or counselling service, general practitioner or family doctor, child and adolescent mental health service, and other mental health professionals.

^3 ^The five belief items were the same as above.

Contrary to the hypotheses, there was no difference in reported help received from teachers or in the students' mental health. A secondary analysis focussing just on students with worse mental health (above the cut-off on the Strengths and Difficulties Questionnaire) at baseline also did not support these hypotheses.

### Adverse events

Given that this was an educational intervention with a non-clinical sample, there was no formal enquiry about adverse events. Informally, no adverse events were reported.

## Discussion

This study showed that the Mental Health First Aid training increased teachers' mental health knowledge, changed beliefs about treatment to be more like those of mental health professionals, reduced some aspects of stigma, and increased confidence in providing help to students and colleagues. These effects were in the small-medium range of effect sizes. Teachers at schools which received the training were also more likely to report that there was a school policy on student mental health and that this policy was implemented. It is impossible to say whether there was an increase in policies being written or whether training gave an increased awareness of existing policies. Most of the changes found in teachers were sustained 6 months after training.

There was an indirect effect on students, who reported receiving more mental health information from their teachers. However, no effects were found on teachers' individual support towards students with mental health problems or on student mental health.

There have been previous trials of Mental Health First Aid training which have found changes in course participants' knowledge, attitudes, confidence and self-reported behaviour [3-10]. However, in these trials it was not possible to study the indirect impact on the recipients of any first aid actions. The only information available on the effects on recipients has been through a qualitative analysis of stories from first aid providers about what had happened to the recipient of their first aid actions [6]. In the present trial, the potential recipients of first aid actions are the students and it was possible to assess any indirect effects on them. No increase in individual student support or change in student mental health was found. However, teachers did become a greater source of mental health information to students. Nevertheless, we cannot rule out the possibility of benefits in support provided to future cohorts of students. The follow-up may have been too short to see any changes in the way teachers assist future students.

The Mental Health First Aid intervention trialled here differs from previous programs involving teachers. Most previous work with teachers has been on school-based prevention of depression and anxiety. Recent reviews have concluded that teacher-administered prevention and early intervention programs are effective for anxiety, but less so for depression [22,23]. These approaches can be seen as complementary to Mental Health First Aid. An intervention that is closer in aims to Mental Health First Aid attempted to improve teachers' recognition of depression in adolescents, but a controlled trial found that this type of training was not successful [24].

This was an effectiveness trial carried out under real-life rather than optimal conditions. It was administered from within the Department of Education and Children's Services, with staff from either the Department or the Child and Adolescent Mental Health Service as instructors. The course content was modified to meet the role expectations of teachers and the duration of the training had to be abbreviated from 14 hours to 7 hours for the majority of staff to fit in with the scheduled staff training days available to schools. Given the modifications and shortening of this course for teachers, the findings do not necessarily apply to the full 14-hour Youth Mental Health First Aid course. Compromises also had to be made in the design of the study. Normally, randomization of schools would occur after baseline assessment. However, this was not feasible because schools needed to know in advance whether they were in the intervention or wait list group so that they could schedule their staff training at the start of the school year. We therefore had to do the pre-test assessment after allocation to groups had occurred. We also had to deal with two schools withdrawing from the project because changes in circumstances did not allow them to do the training as scheduled (e.g. one school got a new principal and the training schedule would have added extra disruption to the changes that this already entailed).

There were a number of significant effects on questionnaire items measuring stigma. However, in part, these changes reflected worsening in the control group as well as improvement in the intervention group. The reason for this pattern of results is unclear, but we speculate that it may be due to social desirability effects with stigma questions. It is possible that participants were biased to give more socially desirable responses at pre-test, but this bias decreased at later assessments. Such effects show the necessity of having a control group to allow for any re-test effects which are unconnected with the intervention.

Although there were many statistically significant findings, these must be viewed in the context of the large number of outcome measures investigated. Clearly, the number of statistically significant findings is greater than expected under the null hypothesis. Leaving out subsidiary analyses of the same variable, 35% for the teacher variables and 9% for the student variables were statistically significant, compared to an expectation of 5%. Some of these effects may be Type I errors. However, it is reassuring that none of the findings were in a direction opposite to that hypothesized (as would be expected with around half of Type I errors) and even the non-significant results often had trends in the expected direction.

## Conclusions

The findings raise issues about the role of teachers in supporting the mental health of their students. In South Australia, educators have an obligation to provide safe and healthy work environments, taking all reasonable measures to eliminate the risk of harm. All workers can access first aid training and provide a basic first aid response. Given the high prevalence of mental health problems in adolescents, it can be argued that teachers need to be able to take action to support students in this area. Just as conventional first aid training and child protection training is considered important, Mental Health First Aid training needs to be considered as a standard component of pre-service or in-service teacher training. However, this training will only be effective if students see teachers as a likely source of initial help for mental health problems. In the present study, only around a quarter of students said they would seek help from a teacher if affected by a mental health problem, compared to around 80% who would seek help from a close family member. Similarly, an Australian national survey of adolescents has found that family and friends are seen as the most important sources of initial help for mental health problems and that teachers do not feature as prominently [11,25]. However, even if students do not see teachers as a first line of help, teachers can still play an important role as a source of mental health information, as the present trial has found. To get optimal benefits for adolescents, it may be necessary to offer Mental Health First Aid training to parents as well as teachers.

## Competing interests

Kitchener and Jorm are developers of the Mental Health First Aid Training Program.

## Authors' contributions

AFJ co-designed the study and took the lead in writing the manuscript. BAK co-designed the study, produced the training curriculum and trained the instructors. MGS co-designed the study. HS coordinated the study and contributed to its design and to the training curriculum. SC carried out the statistical analysis. All authors read and approved the final manuscript.

## Pre-publication history

The pre-publication history for this paper can be accessed here:

http://www.biomedcentral.com/1471-244X/10/51/prepub

## Supplementary Material

Additional file 1 **More detailed analyses of teacher outcome variables**. Click here for file

Additional file 2 **More detailed analyses of student outcome variables**. Click here for file
