# Supplementary material for: Mental health first aid training for high school teachers: a cluster randomized trial
Source: BMC Psychiatry. 2010 Jun 24;10:51. doi: 10.1186/1471-244X-10-51 (PMC2908569; doi:10.1186/1471-244X-10-51)
Supplement: Additional file 1 — More detailed analyses of teacher outcome variables. [file 1471-244X-10-51-S1.DOCX]

### Additional file 1 – More detailed analyses of teacher outcome variables

**Table 1: Analyses of teacher outcome variables – 14 randomised schools**

|  | **Intervention group** | | |  | **Control group** | | |  | **Mean diff./OR for pre vs post by intervention interaction (95% CI)** | **Mean diff./OR for pre vs follow-up by intervention interaction (95% CI)** |
| --- | --- | --- | --- | --- | --- | --- | --- | --- | --- | --- |
|  | **Pre** | **Post** | **Follow-up** |  | **Pre** | **Post** | **Follow-up** |  |  |  |
| **Mental Health Knowledge** |  |  |  |  |  |  |  |  |  |  |
| Knowledge quiz: mean (SD) | 11.14 (3.57) | 13.07 (3.30) | 12.68 (3.44) |  | 11.26 (3.07) | 11.11 (3.58) | 10.76 (3.89) |  | 2.08 (1.38-2.78)*** | 1.79 (1.06-2.52)*** |
| Recognition of depression % | 81.8 | 86.1 | 92.9 |  | 80.6 | 85.9 | 83.8 |  | 0.98 (0.27-3.56) | 3.09 (0.77-12.43) |
| Beliefs about treatment for depression: mean (SD) | 8.22 (2.39) | 8.85 (2.54) | 8.86 (2.39) |  | 7.91 (2.44) | 7.84 (2.74) | 7.92 (2.46) |  | 0.79 (0.23-1.34)** | 0.73 (0.15-1.31)* |
| *Separate items for beliefs about treatment for depression: % helpful* |  |  |  |  |  |  |  |  |  |  |
| ·         Becoming physically active | 69.3 | 79.4 | 84.8 |  | 67.9 | 76.0 | 76.7 |  | 1.43 (0.52-3.97) | 2.04 (0.69-6.00) |
| ·         Getting relaxation training | 81.2 | 88.2 | 89.9 |  | 81.9 | 80.6 | 86.1 |  | 2.95 (0.91-9.63) | 2.08 (0.59-7.42) |
| ·         Receiving counselling | 93.6 | 93.5 | 94.3 |  | 93.3 | 86.6 | 82.8 |  | 2.90 (0.62-13.52) | 6.65 (1.31-33.92)* |
| ·         Receiving CBT | 39.2 | 54.7 | 47.8 |  | 36.5 | 30.6 | 27.9 |  | 6.64 (2.15-20.53)** | 4.43 (1.37-14.29)* |
| ·         Local mental health service | 69.7 | 81.1 | 79.3 |  | 68.3 | 69.4 | 63.2 |  | 2.42 (0.92-6.36) | 2.90 (1.09-7.71)* |
| ·         School/student counsellor | 90.4 | 91.8 | 93.1 |  | 91.5 | 88.8 | 90.8 |  | 2.25 (0.50-10.16) | 2.23 (0.45-11.06) |
| ·         Telephone counselling service | 90.9 | 90.1 | 92.5 |  | 86.8 | 89.8 | 87.4 |  | 0.64 (0.16-2.61) | 1.48 (0.34-6.41) |
| ·         GP or family doctor | 85.5 | 91.2 | 93.1 |  | 87.7 | 89.7 | 90.8 |  | 1.81 (0.48-6.83) | 2.18 (0.52-9.17) |
| ·         Psychologist | 72.3 | 77.7 | 76.1 |  | 64.2 | 63.3 | 64.4 |  | 1.83 (0.65-5.12) | 1.38 (0.47-4.00) |
| ·         Psychiatrist | 53.6 | 60.2 | 57.2 |  | 50.0 | 44.9 | 54.0 |  | 2.14 (0.86-5.33) | 1.02 (0.40-2.61) |
| ·         Other mental health professionals | 75.0 | 73.1 | 76.0 |  | 63.8 | 67.4 | 60.9 |  | 0.70 (0.28-1.79) | 1.29 (0.49-3.36) |
| **Personal Stigma Items: % Strongly Disagree** |  |  |  |  |  |  |  |  |  |  |
| Could snap out of it | 32.1 | 40.1 | 37.3 |  | 31.1 | 29.6 | 26.4 |  | 2.12 (0.76-5.90) | 2.59 (0.87-7.69) |
| Personal weakness | 53.9 | 54.4 | 55.4 |  | 63.2 | 49.0 | 54.0 |  | 3.07 (1.16-8.14)* | 2.47 (0.91-6.76) |
| Not real illness: % | 45.0 | 47.1 | 48.7 |  | 43.4 | 37.8 | 34.5 |  | 1.70 (0.67-4.32) | 2.50 (0.94-6.66) |
| People with that problem are dangerous | 35.6 | 37.7 | 38.0 |  | 35.2 | 34.7 | 33.3 |  | 1.05 (0.39-2.82) | 1.60 (0.57-4.45) |
| Best to avoid people with that problem | 72.3 | 62.0 | 66.0 |  | 68.9 | 62.2 | 59.8 |  | 0.75 (0.30-1.89) | 1.17 (0.45-3.03) |
| People with that problem are unpredictable | 8.1 | 12.3 | 12.7 |  | 14.2 | 10.4 | 11.5 |  | 3.54 (0.88-14.17) | 3.36 (0.82-13.83) |
| If they had problem they would not tell anyone | 25.0 | 31.4 | 26.4 |  | 28.3 | 18.6 | 16.1 |  | 3.79 (1.34-10.71)* | 3.42 (1.13-10.32)* |
| *Alternative coding of personal stigma items: % ≥ disagree* |  |  |  |  |  |  |  |  |  |  |
| ·         Could snap out of it | 81.2 | 85.5 | 86.7 |  | 84.9 | 83.7 | 78.2 |  | 1.92 (0.54-6.80) | 4.92 (1.33-18.24)* |
| ·         Personal weakness | 95.4 | 91.2 | 93.1 |  | 94.3 | 91.8 | 92.0 |  | 0.59 (0.08-4.30) | 1.39 (0.17-11.29) |
| ·         Not real illness | 84.6 | 90.1 | 91.8 |  | 89.6 | 87.8 | 82.8 |  | 3.35 (0.83-13.55) | 7.88 (1.85-33.6)** |
| ·         People with that problem are dangerous | 76.7 | 79.4 | 86.1 |  | 81.0 | 83.7 | 82.8 |  | 0.93 (0.29-2.93) | 2.38 (0.71-8.00) |
| ·         Best to avoid people with that problem | 95.5 | 95.9 | 96.9 |  | 97.2 | 95.9 | 95.4 |  | 1.63 (0.26-10.08) | 2.40 (0.36-15.76) |
| ·         People with that problem are unpredictable | 42.5 | 43.3 | 41.8 |  | 50.0 | 49.0 | 51.7 |  | 1.15 (0.49-2.70) | 0.85 (0.35-2.04) |
| ·         If they had problem they would not tell anyone | 70.5 | 73.8 | 74.8 |  | 72.6 | 74.2 | 66.7 |  | 1.06 (0.39-2.89) | 2.17 (0.79-6.01) |
| **Perceived Stigma Items: % ≥ Agree** |  |  |  |  |  |  |  |  |  |  |
| Other people think could snap out of it | 64.6 | 57.0 | 57.2 |  | 64.8 | 59.8 | 54.7 |  | 0.88 (0.34-2.26) | 1.24 (0.47-3.33) |
| Other people believe a sign of personal weakness | 52.7 | 52.9 | 56.0 |  | 58.5 | 56.7 | 45.9 |  | 1.10 (0.42-2.87) | 3.01 (1.10-8.23)* |
| Other people believe not real illness | 62.4 | 55.8 | 59.8 |  | 60.4 | 55.7 | 57.0 |  | 0.86 (0.37-2.02) | 1.07 (0.44-2.60) |
| Other people believe they are dangerous | 19.1 | 25.0 | 25.2 |  | 26.4 | 20.6 | 22.1 |  | 2.75 (0.98-7.66) | 2.05 (0.72-5.85) |
| Other people would avoid people with that problem | 23.6 | 29.7 | 28.9 |  | 27.4 | 23.7 | 24.4 |  | 2.42 (0.85-6.87) | 1.90 (0.65-5.54) |
| Other people believe they are unpredictable | 53.6 | 50.6 | 51.6 |  | 45.2 | 46.9 | 45.4 |  | 0.72 (0.31-1.68) | 0.95 (0.40-2.28) |
| Other people would not tell anyone | 61.4 | 59.1 | 51.6 |  | 67.6 | 51.6 | 52.9 |  | 2.57 (1.04-6.35)* | 1.32 (0.52-3.36) |
| **Intended Helping Behaviours Towards Students** |  |  |  |  |  |  |  |  |  |  |
| Contact the family: % *≥ often* | 38.2 | 41.8 | 44.0 |  | 36.2 | 37.5 | 35.3 |  | 1.28 (0.47-3.48) | 1.46 (0.52-4.13) |
| Discuss with another teacher: % *≥ often* | 72.3 | 80.1 | 73.4 |  | 69.5 | 62.9 | 60.7 |  | 3.73 (1.31-10.62)* | 2.46 (0.86-7.05) |
| Discuss with counsellors: % *≥ often* | 82.3 | 87.1 | 86.6 |  | 81.9 | 74.5 | 75.9 |  | 3.87 (1.21-12.41)* | 2.98 (0.90-9.91) |
| Discuss with member of administration: % *≥ often* | 37.7 | 39.2 | 40.8 |  | 42.9 | 39.8 | 47.1 |  | 1.36 (0.52-3.60) | 0.99 (0.37-2.68) |
| Have conversation with student: % *≥ often* | 68.6 | 72.5 | 70.3 |  | 61.0 | 58.2 | 49.4 |  | 2.06 (0.75-5.68) | 3.16 (1.10-9.06)* |
| Talk with peers of student: % *≥ often* | 18.2 | 22.2 | 21.0 |  | 13.6 | 9.2 | 12.6 |  | 3.24 (0.91-11.54) | 1.70 (0.49-5.94) |
| Do nothing: % *never* | 65.5 | 66.1 | 66.5 |  | 69.5 | 65.0 | 61.6 |  | 1.95 (0.70-5.48) | 2.37 (0.82-6.81) |
| **Help Given Towards Students: % ≥ Occasionally** |  |  |  |  |  |  |  |  |  |  |
| Spoken with students about their mental health problems | 52.1 | 52.1 | 54.8 |  | 53.3 | 51.0 | 47.7 |  | 1.34 (0.48-3.75) | 1.73 (0.59-5.08) |
| *If spoken with students about their mental health problems, response to bullet point items below:* |  |  |  |  |  |  |  |  |  |  |
| ·         Spent time listening to problem | 86.0 | 90.8 | 84.8 |  | 85.9 | 88.7 | 80.7 |  | 2.44 (0.39-15.17) | 2.36 (0.42-13.37) |
| ·         Helped to calm them down | 77.3 | 74.0 | 74.1 |  | 69.0 | 74.2 | 73.7 |  | 0.47 (0.13-1.72) | 0.51 (0.13-1.94) |
| ·         Talked to them about suicidal thoughts | 20.8 | 22.9 | 24.8 |  | 15.7 | 11.5 | 17.2 |  | 1.88 (0.39-9.20) | 1.14 (0.24-5.37) |
| ·         Recommended they seek professional help | 50.7 | 53.0 | 52.3 |  | 57.1 | 40.0 | 41.4 |  | 4.09 (1.19-14.12)* | 3.16 (0.90-11.01) |
| ·         Something else | 47.2 | 47.8 | 54.6 |  | 76.5 | 66.7 | 50.0 |  | *Numbers too small – ORs not meaningful* | |
| Discussed a students’ mental health problems with other teachers | 67.9 | 72.4 | 66.2 |  | 70.5 | 68.4 | 58.1 |  | 1.87 (0.67-5.22) | 1.91 (0.68-5.41) |
| Mental health issues raised in staff meetings | 57.9 | 50.3 | 47.1 |  | 62.1 | 52.6 | 47.7 |  | 1.26 (0.51-3.07) | 1.22 (0.48-3.08) |
| **Confidence in Helping Students and Staff with Mental Health Problems: % ≥ Quite a Bit** |  |  |  |  |  |  |  |  |  |  |
| Confidence to talk with students about mental health problems | 19.0 | 32.6 | 34.2 |  | 20.8 | 20.4 | 17.4 |  | 8.09 (1.89-34.63)** | 7.02 (1.65-29.79)** |
| Confidence in helping a colleague with mental health problem | 16.4 | 25.0 | 32.3 |  | 20.8 | 15.3 | 14.9 |  | 7.22 (1.84-28.4)** | 11.65 (2.87-47.32)*** |
| **School Policies on Student Mental Health** |  |  |  |  |  |  |  |  |  |  |
| Review curriculum options/classroom practices: % *≥ often* | 54.3 | 56.7 | 58.0 |  | 59.1 | 48.5 | 41.9 |  | 2.22 (0.93-5.26) | 3.76 (1.51-9.34)** |
| Review/changes school policy: % *≥ often* | 18.6 | 24.1 | 21.2 |  | 20.4 | 12.4 | 12.9 |  | 3.20 (1.12-9.14)* | 2.44 (0.82-7.26) |
| Set up planned family liaison: % *≥ often* | 59.2 | 62.4 | 61.8 |  | 58.1 | 51.6 | 47.7 |  | 1.86 (0.78-4.40) | 2.56 (1.04-6.27)* |
| Set up planned community liaison: % *≥ often* | 33.6 | 38.2 | 39.5 |  | 32.4 | 27.8 | 25.6 |  | 1.90 (0.75-4.84) | 2.81 (1.05-7.52)* |
| External support for student and family: *≥ often* | 58.5 | 61.0 | 56.4 |  | 55.2 | 51.0 | 40.7 |  | 1.57 (0.65-3.80) | 2.36 (0.93-6.00) |
| Improve the relationships within the school: % *≥ often* | 65.6 | 69.4 | 68.2 |  | 71.4 | 61.2 | 58.1 |  | 3.09 (1.12-8.52)* | 3.26 (1.14-9.27)* |
| School has written policy to deal with students with mental health problems: % *yes* | 10.1 | 22.7 | 28.5 |  | 11.5 | 11.2 | 10.5 |  | 4.57 (1.28-16.26)* | 7.28 (1.92-27.54)** |
| Policy been implemented in the last month: % *≥ occasionally* | 9.8 | 14.2 | 17.8 |  | 13.4 | 7.0 | 11.3 |  | 7.23 (0.85-61.37) | 13.30 (1.32-133.44)* |
| **School Actions** |  |  |  |  |  |  |  |  |  |  |
| Contact the family: % *≥ often* | 76.5 | 74.3 | 75.3 |  | 69.2 | 76.3 | 67.4 |  | 0.55 (0.22-1.42) | 1.07 (0.41-2.76) |
| Remind all students of school policies/services/rules: % *≥ often* | 37.8 | 34.7 | 36.5 |  | 31.0 | 36.1 | 30.6 |  | 0.57 (0.24-1.39) | 0.88 (0.35-2.22) |
| Classroom teacher conference to plan student support: % *≥ often* | 51.4 | 48.5 | 50.0 |  | 45.2 | 55.2 | 41.9 |  | 0.51 (0.22-1.20) | 1.27 (0.53-3.09) |
| Behaviour consequences: % *≥ often* | 10.2 | 9.4 | 5.7 |  | 9.7 | 9.3 | 7.0 |  | 0.96 (0.26-3.58) | 0.76 (0.17-3.36) |
| Mediation: % *≥ often* | 66.8 | 71.8 | 62.0 |  | 57.7 | 58.8 | 53.5 |  | 1.32 (0.57-3.05) | 1.01 (0.43-2.35) |
| Referral to outside agency: % *≥ often* | 44.7 | 51.8 | 44.3 |  | 46.2 | 38.1 | 43.0 |  | 2.70 (1.09-6.67)* | 1.16 (0.46-2.91) |
| Do nothing: % *never* | 59.3 | 64.1 | 62.0 |  | 60.0 | 59.8 | 59.3 |  | 1.59 (0.64-3.95) | 1.27 (0.50-3.25) |
| **Interacting with Colleagues: % ≥ Occasionally** |  |  |  |  |  |  |  |  |  |  |
| Talked with staff member about their mental health problem | 39.1 | 38.0 | 38.3 |  | 38.4 | 38.1 | 36.1 |  | 0.88 (0.35-2.22) | 0.93 (0.35-2.45) |
| *If talked with staff member about their mental health problem, response to bullet point items below* |  |  |  |  |  |  |  |  |  |  |
| ·         Spent time listening to their problem | 80.2 | 81.6 | 75.8 |  | 81.0 | 83.0 | 74.5 |  | 1.11 (0.17-7.44) | 1.03 (0.17-6.37) |
| ·         Helped them calm down | 67.3 | 66.7 | 55.1 |  | 66.7 | 64.4 | 58.7 |  | 2.12 (0.43-10.56) | 0.89 (0.19-4.08) |
| ·         Talked to them about suicidal thoughts | 15.9 | 13.8 | 11.2 |  | 7.0 | 2.2 | 6.5 |  | 3.73 (0.24-57.11) | 0.60 (0.08-4.82) |
| ·         Recommended they seek professional help | 43.0 | 40.2 | 29.6 |  | 24.6 | 21.7 | 23.9 |  | 1.04 (0.23-4.75) | 0.37 (0.08-1.71) |
| ·         Something else | 30.0 | 50.0 | 18.8 |  | 71.4 | 66.7 | 28.6 |  | *Numbers too small – ORs not meaningful* | |
| Talk about own mental health problem with a staff member | 35.8 | 39.4 | 38.2 |  | 37.1 | 34.7 | 34.5 |  | 1.49 (0.58-3.82) | 1.23 (0.46-3.29) |
| **Seeking Additional Mental Health Information: % ≥ Occasionally** |  |  |  |  |  |  |  |  |  |  |
| Visit any websites giving information about mental health | 21.8 | 23.5 | 26.8 |  | 21.0 | 19.6 | 17.2 |  | 1.29 (0.42-3.91) | 1.81 (0.56-5.79) |
| Read books or other written material bout mental health problems | 43.9 | 49.1 | 39.9 |  | 38.1 | 38.8 | 35.6 |  | 1.30 (0.51-3.34) | 0.85 (0.31-2.31) |
| **Teacher Mental Health** |  |  |  |  |  |  |  |  |  |  |
| K6 6-24 (severe psychological distress) % | 29.8 | 34.3 | 25.8 |  | 25.5 | 22.1 | 25.3 |  | 2.41 (0.77-7.49) | 0.66 (0.20-2.13) |
| K6 3-24 (medium-high psychological distress) % | 63.5 | 59.2 | 58.9 |  | 58.8 | 55.8 | 59.0 |  | 0.96 (0.34-2.70) | 0.61 (0.20-1.85) |
| *Restricted case analyses of K6* |  |  |  |  |  |  |  |  |  |  |
| ·         Analysis 1 K6 6-24^1^% | 21.6 | 23.7 | 20.5 |  | 15.7 | 16.8 | 14.5 |  | 1.01 (0.00-312.65) | 1.02 (0.00-563.45) |
| ·         Analysis 2 K6 6-24^2^ % | 24.9 | 28.6 | 24.6 |  | 18.2 | 19.8 | 16.9 |  | 1.04 (0.00-307.32) | 1.04 (0.00-543.66) |
| ·         Analysis 1 K6 3-24^1^ % | 50.0 | 53.3 | 53.6 |  | 48.0 | 50.5 | 50.6 |  | 1.01 (0.04-25.75) | 1.03 (0.03-31.39) |
| ·         Analysis 2 K6 3-24^2^ % | 55.9 | 62.9 | 64.3 |  | 54.4 | 59.3 | 57.5 |  | 1.27 (0.03-55.18) | 1.40 (0.03-71.92) |

Legend: * p<0.05; ** p<0.01; *** p<0.001

^1^ The outcomes of teachers that scored 6-24 (or 3-24) on the K6 at assessment 1.

^2^ The outcomes of teachers that scored 6-24 (or 3-24) on the K6 at assessment 1, but also excludes teachers that crossed over into the 6-24 (or 3-24) range at assessment 2 and/or 3.

**Table 2: Analyses of teacher outcome variables – 16 schools**

|  | **Intervention group** | | |  | **Control group** | | |  | **Mean diff./OR for pre vs post by intervention interaction (95% CI)** | **Mean diff./OR for pre vs follow-up by intervention interaction (95% CI)** |
| --- | --- | --- | --- | --- | --- | --- | --- | --- | --- | --- |
|  | **Pre** | **Post** | **Follow-up** |  | **Pre** | **Post** | **Follow-up** |  |  |  |
| **Mental Health Knowledge** |  |  |  |  |  |  |  |  |  |  |
| Knowledge quiz: mean (SD) | 11.08 (3.54) | 12.91 (3.32) | 12.67 (3.42) |  | 11.39 (3.15) | 11.23 (3.50) | 10.99 (3.89) |  | 1.99 (1.33-2.64)*** | 1.79 (1.11-2.48)*** |
| Recognition of depression % | 82.7 | 87.3 | 92.3 |  | 80.9 | 86.3 | 83.0 |  | 0.97 (0.28-3.34) | 2.94 (0.78-11.00) |
| Beliefs about treatment for depression: mean (SD) | 8.17 (2.37) | 8.79 (2.44) | 8.79 (2.45) |  | 7.90 (2.48) | 7.90 (2.70) | 7.98 (2.44) |  | 0.71 (0.19-1.24)** | 0.58 (0.04-1.13)* |
| *Separate items for beliefs about treatment for depression: % helpful* |  |  |  |  |  |  |  |  |  |  |
| ·         Becoming physically active | 69.4 | 79.0 | 82.2 |  | 67.0 | 75.7 | 76.8 |  | 1.28 (0.50-3.29) | 1.38 (0.51-3.74) |
| ·         Getting relaxation training | 81.6 | 89.2 | 89.7 |  | 81.2 | 82.6 | 85.3 |  | 2.42 (0.80-7.36) | 1.94 (0.60-6.32) |
| ·         Receiving counselling | 93.9 | 93.9 | 93.1 |  | 92.2 | 88.0 | 83.3 |  | 1.91 (0.46-8.01) | 3.22 (0.75-13.89) |
| ·         Receiving CBT | 38.1 | 53.3 | 45.1 |  | 38.8 | 33.0 | 29.5 |  | 6.40 (2.23-18.34)*** | 4.29 (1.42-12.97)** |
| ·         Local mental health service | 68.4 | 80.4 | 78.9 |  | 67.2 | 67.9 | 63.5 |  | 2.54 (1.04-6.23)* | 2.71 (1.08-6.79)* |
| ·         School/student counsellor | 90.7 | 91.8 | 93.1 |  | 90.7 | 88.1 | 91.7 |  | 2.10 (0.50-8.76) | 1.60 (0.35-7.41) |
| ·         Telephone counselling service | 90.7 | 91.3 | 92.6 |  | 86.4 | 89.9 | 86.5 |  | 0.74 (0.19-2.89) | 1.64 (0.40-6.72) |
| ·         GP or family doctor | 86.6 | 90.8 | 93.1 |  | 87.3 | 88.9 | 91.7 |  | 1.51 (0.44-5.12) | 1.49 (0.38-5.86) |
| ·         Psychologist | 70.5 | 75.9 | 76.0 |  | 64.4 | 63.3 | 64.6 |  | 1.91 (0.71-5.09) | 1.48 (0.53-4.16) |
| ·         Psychiatrist | 52.2 | 57.7 | 57.7 |  | 50.9 | 46.8 | 55.2 |  | 1.86 (0.78-4.45) | 1.07 (0.43-2.66) |
| ·         Other mental health professionals | 73.3 | 73.0 | 75.3 |  | 65.0 | 67.9 | 63.5 |  | 0.83 (0.34-2.00) | 1.21 (0.48-3.01) |
| **Personal Stigma Items: % Strongly Disagree** |  |  |  |  |  |  |  |  |  |  |
| Could snap out of it | 30.6 | 38.1 | 36.8 |  | 32.2 | 29.4 | 27.1 |  | 2.32 (0.87-6.21) | 3.07 (1.07-8.75)* |
| Personal weakness | 52.0 | 55.1 | 54.9 |  | 64.4 | 48.6 | 52.1 |  | 4.21 (1.65-10.76)** | 3.41 (1.30-9.00)* |
| Not real illness: % | 43.3 | 47.2 | 47.7 |  | 44.1 | 38.5 | 34.4 |  | 1.93 (0.79-4.74) | 2.77 (1.07-7.20)* |
| People with that problem are dangerous | 34.6 | 36.9 | 37.9 |  | 35.0 | 34.9 | 31.3 |  | 1.04 (0.40-2.66) | 2.03 (0.75-5.51) |
| Best to avoid people with that problem | 70.9 | 62.8 | 63.4 |  | 70.3 | 63.3 | 59.4 |  | 0.91 (0.38-2.18) | 1.25 (0.50-3.10) |
| People with that problem are unpredictable | 8.9 | 11.7 | 12.1 |  | 13.6 | 10.3 | 11.5 |  | 2.69 (0.72-10.04) | 2.60 (0.67-10.04) |
| If they had problem they would not tell anyone | 25.6 | 30.0 | 25.7 |  | 30.5 | 18.5 | 16.7 |  | 3.66 (1.39-9.65)** | 3.40 (1.21-9.56)* |
| *Alternative coding of personal stigma items: % ≥ disagree* |  |  |  |  |  |  |  |  |  |  |
| ·         Could snap out of it | 80.8 | 86.3 | 86.2 |  | 86.4 | 84.4 | 79.2 |  | 2.43 (0.74-7.92) | 4.89 (1.44-16.63)* |
| ·         Personal weakness | 95.5 | 91.8 | 93.1 |  | 94.9 | 92.7 | 92.7 |  | 0.63 (0.09-4.43) | 1.21 (0.15-9.44) |
| ·         Not real illness | 84.2 | 91.4 | 90.8 |  | 90.7 | 87.2 | 82.3 |  | 5.49 (1.43-21.02)* | 8.48 (2.15-33.49)** |
| ·         People with that problem are dangerous | 76.4 | 79.5 | 85.1 |  | 81.2 | 84.4 | 83.3 |  | 0.92 (0.31-2.76) | 2.11 (0.67-6.71) |
| ·         Best to avoid people with that problem | 95.1 | 96.4 | 96.0 |  | 97.5 | 96.3 | 95.8 |  | 2.04 (0.33-12.66) | 2.02 (0.32-12.63) |
| ·         People with that problem are unpredictable | 41.5 | 45.4 | 41.4 |  | 47.5 | 48.6 | 51.0 |  | 1.23 (0.54-2.79) | 0.80 (0.34-1.86) |
| ·         If they had problem they would not tell anyone | 71.1 | 74.6 | 74.9 |  | 72.9 | 75.0 | 66.7 |  | 0.97 (0.37-2.56) | 2.25 (0.84-6.02) |
| **Perceived Stigma Items: % ≥ Agree** |  |  |  |  |  |  |  |  |  |  |
| Other people think could snap out of it | 64.0 | 55.9 | 56.0 |  | 65.0 | 63.0 | 53.7 |  | 0.69 (0.29-1.65) | 1.22 (0.49-3.03) |
| Other people believe a sign of personal weakness | 51.0 | 52.3 | 54.9 |  | 59.3 | 59.3 | 45.7 |  | 1.08 (0.43-2.71) | 3.28 (1.25-8.59)* |
| Other people believe not real illness | 60.8 | 54.4 | 57.1 |  | 58.5 | 58.3 | 55.8 |  | 0.67 (0.30-1.48) | 0.90 (0.39-2.07) |
| Other people believe they are dangerous | 19.0 | 23.6 | 25.7 |  | 27.1 | 25.0 | 23.2 |  | 1.75 (0.68-4.49) | 2.00 (0.75-5.36) |
| Other people would avoid people with that problem | 24.7 | 28.7 | 29.1 |  | 28.8 | 26.9 | 25.3 |  | 1.73 (0.65-4.57) | 1.64 (0.60-4.52) |
| Other people believe they are unpredictable | 52.6 | 51.3 | 49.7 |  | 47.4 | 50.5 | 46.3 |  | 0.74 (0.33-1.64) | 0.93 (0.41-2.13) |
| Other people would not tell anyone | 60.3 | 57.2 | 50.3 |  | 66.7 | 53.7 | 52.1 |  | 1.98 (0.85-4.64) | 1.21 (0.50-2.92) |
| **Intended Helping Behaviours Towards Students** |  |  |  |  |  |  |  |  |  |  |
| Contact the family: % *≥ often* | 38.1 | 42.1 | 43.4 |  | 39.3 | 39.3 | 36.2 |  | 1.49 (0.59-3.73) | 1.80 (0.68-4.72) |
| Discuss with another teacher: % *≥ often* | 70.5 | 78.6 | 70.7 |  | 68.4 | 63.0 | 59.1 |  | 3.43 (1.30-9.08)* | 2.25 (0.84-5.98) |
| Discuss with counsellors: % *≥ often* | 82.5 | 86.7 | 85.6 |  | 82.1 | 76.2 | 77.1 |  | 3.17 (1.06-9.54)* | 2.36 (0.75-7.36) |
| Discuss with member of administration: % *≥ often* | 38.5 | 39.0 | 39.9 |  | 42.7 | 43.1 | 46.9 |  | 0.96 (0.39-2.39) | 0.88 (0.34-2.25) |
| Have conversation with student: % *≥ often* | 65.2 | 68.9 | 69.0 |  | 64.1 | 59.6 | 51.0 |  | 2.39 (0.90-6.33) | 4.18 (1.50-11.62)** |
| Talk with peers of student: % *≥ often* | 17.8 | 21.4 | 19.7 |  | 16.5 | 11.0 | 14.6 |  | 3.48 (1.08-11.25)* | 1.71 (0.53-5.51) |
| Do nothing: % *never* | 64.4 | 65.8 | 63.8 |  | 71.8 | 65.7 | 64.2 |  | 2.39 (0.89-6.43) | 1.99 (0.72-5.48) |
| **Help Given Towards Students: % ≥ Occasionally** |  |  |  |  |  |  |  |  |  |  |
| Spoken with students about their mental health problems | 51.6 | 51.0 | 55.8 |  | 56.4 | 52.3 | 50.5 |  | 1.50 (0.57-3.96) | 1.85 (0.67-5.09) |
| *If spoken with students about their mental health problems, response to bullet point items below:* |  |  |  |  |  |  |  |  |  |  |
| ·         Spent time listening to problem | 86.1 | 89.8 | 84.9 |  | 87.7 | 88.6 | 81.3 |  | 2.18 (0.42-11.33) | 2.28 (0.47-11.14) |
| ·         Helped to calm them down | 77.6 | 75.6 | 75.4 |  | 72.8 | 75.7 | 75.0 |  | 0.62 (0.18-2.11) | 0.63 (0.18-2.25) |
| ·         Talked to them about suicidal thoughts | 19.5 | 21.6 | 22.8 |  | 18.8 | 13.0 | 18.5 |  | 2.30 (0.53-9.89) | 1.32 (0.31-5.54) |
| ·         Recommended they seek professional help | 49.1 | 50.0 | 48.8 |  | 59.5 | 39.7 | 44.6 |  | 4.45 (1.41-14.01)* | 2.54 (0.80-8.08) |
| ·         Something else | 48.7 | 44.4 | 53.9 |  | 70.0 | 62.5 | 46.2 |  | *Numbers too small – ORs not meaningful* | |
| Discussed a students’ mental health problems with other teachers | 67.2 | 69.6 | 65.9 |  | 70.9 | 69.7 | 59.0 |  | 1.41 (0.54-3.66) | 1.82 (0.69-4.80) |
| Mental health issues raised in staff meetings | 57.1 | 48.4 | 47.1 |  | 61.7 | 54.6 | 46.3 |  | 1.00 (0.44-2.29) | 1.37 (0.57-3.26) |
| **Confidence in Helping Students and Staff with Mental Health Problems: % ≥ Quite a Bit** |  |  |  |  |  |  |  |  |  |  |
| Confidence to talk with students about mental health problems | 18.6 | 32.7 | 32.2 |  | 22.9 | 20.2 | 19.0 |  | 11.68 (2.96-46.13)*** | 5.80 (1.54-21.79)** |
| Confidence in helping a colleague with mental health problem | 16.6 | 26.0 | 30.5 |  | 20.3 | 14.7 | 15.6 |  | 7.58 (2.09-27.47)** | 7.51 (2.04-27.62)** |
| **School Policies on Student Mental Health** |  |  |  |  |  |  |  |  |  |  |
| Review curriculum options/classroom practices: % *≥ often* | 54.9 | 57.1 | 56.1 |  | 59.8 | 45.4 | 42.1 |  | 2.83 (1.25-6.42)* | 3.32 (1.40-7.87)** |
| Review/changes school policy: % *≥ often* | 18.6 | 23.1 | 20.4 |  | 19.1 | 11.1 | 12.8 |  | 3.13 (1.13-8.63)* | 2.07 (0.73-5.88) |
| Set up planned family liaison: % *≥ often* | 59.6 | 60.5 | 59.5 |  | 60.7 | 55.6 | 50.5 |  | 1.53 (0.67-3.52) | 2.23 (0.94-5.31) |
| Set up planned community liaison: % *≥ often* | 34.0 | 37.6 | 37.6 |  | 33.3 | 33.3 | 28.4 |  | 1.30 (0.55-3.06) | 2.02 (0.81-5.06) |
| External support for student and family: *≥ often* | 57.7 | 60.6 | 56.4 |  | 57.3 | 55.1 | 43.2 |  | 1.48 (0.63-3.48) | 2.44 (0.99-6.03) |
| Improve the relationships within the school: % *≥ often* | 64.5 | 69.1 | 65.9 |  | 70.9 | 61.5 | 61.1 |  | 3.23 (1.24-8.40)* | 2.32 (0.86-6.22) |
| School has written policy to deal with students with mental health problems: % *yes* | 9.8 | 21.9 | 27.6 |  | 10.3 | 11.0 | 10.5 |  | 3.76 (1.13-12.58)* | 5.69 (1.61-20.11)** |
| Policy been implemented in the last month: % *≥ occasionally* | 8.6 | 13.3 | 16.8 |  | 12.9 | 7.5 | 11.4 |  | 7.11 (1.04-48.76)* | 10.90 (1.41-84.48)* |
| **School Actions** |  |  |  |  |  |  |  |  |  |  |
| Contact the family: % *≥ often* | 75.8 | 74.0 | 74.7 |  | 71.6 | 77.8 | 69.5 |  | 0.59 (0.24-1.45) | 1.11 (0.45-2.77) |
| Remind all students of school policies/services/rules: % *≥ often* | 37.3 | 34.9 | 35.5 |  | 33.0 | 36.1 | 30.9 |  | 0.70 (0.31-1.62) | 0.93 (0.39-2.24) |
| Classroom teacher conference to plan student support: % *≥ often* | 51.4 | 48.5 | 47.7 |  | 47.4 | 55.1 | 43.2 |  | 0.59 (0.26-1.32) | 1.18 (0.51-2.72) |
| Behaviour consequences: % *≥ often* | 10.3 | 9.2 | 5.2 |  | 10.4 | 9.3 | 6.3 |  | 1.04 (0.30-3.54) | 0.82 (0.19-3.47) |
| Mediation: % *≥ often* | 66.0 | 70.3 | 59.2 |  | 58.6 | 61.1 | 53.7 |  | 1.19 (0.54-2.62) | 0.93 (0.42-2.07) |
| Referral to outside agency: % *≥ often* | 44.7 | 50.5 | 42.2 |  | 47.4 | 40.7 | 44.2 |  | 2.24 (0.96-5.19) | 0.98 (0.41-2.35) |
| Do nothing: % *never* | 59.3 | 62.6 | 61.5 |  | 61.5 | 58.3 | 60.0 |  | 1.73 (0.74-4.04) | 1.31 (0.54-3.18) |
| **Interacting with Colleagues: % ≥ Occasionally** |  |  |  |  |  |  |  |  |  |  |
| Talked with staff member about their mental health problem | 38.1 | 36.7 | 39.1 |  | 38.7 | 38.0 | 36.3 |  | 0.90 (0.38-2.17) | 1.07 (0.43-2.69) |
| *If talked with staff member about their mental health problem, response to bullet point items below* |  |  |  |  |  |  |  |  |  |  |
| ·         Spent time listening to their problem | 80.5 | 77.8 | 76.2 |  | 81.3 | 83.0 | 70.4 |  | 0.76 (0.15-3.93) | 1.51 (0.32-7.12) |
| ·         Helped them calm down | 66.9 | 62.6 | 56.5 |  | 68.3 | 64.7 | 54.7 |  | 1.69 (0.39-7.36) | 1.34 (0.33-5.48) |
| ·         Talked to them about suicidal thoughts | 14.6 | 12.1 | 11.1 |  | 9.5 | 1.9 | 5.7 |  | 6.46 (0.48-86.55) | 1.40 (0.20-9.58) |
| ·         Recommended they seek professional help | 42.4 | 39.4 | 29.6 |  | 27.0 | 19.2 | 24.5 |  | 1.61 (0.40-6.47) | 0.49 (0.12-1.91) |
| ·         Something else | 27.3 | 47.4 | 16.7 |  | 73.3 | 40.0 | 22.2 |  | *Numbers too small – ORs not meaningful* | |
| Talk about own mental health problem with a staff member | 35.7 | 39.0 | 37.6 |  | 40.2 | 33.9 | 34.4 |  | 1.91 (0.78-4.66) | 1.42 (0.56-3.60) |
| **Seeking Additional Mental Health Information: % ≥ Occasionally** |  |  |  |  |  |  |  |  |  |  |
| Visit any websites giving information about mental health | 21.1 | 24.1 | 25.4 |  | 23.9 | 20.4 | 18.8 |  | 1.82 (0.63-5.20) | 1.97 (0.65-5.95) |
| Read books or other written material bout mental health problems | 43.7 | 49.0 | 38.5 |  | 41.0 | 40.4 | 37.5 |  | 1.45 (0.59-3.57) | 0.82 (0.32-2.16) |
| **Teacher Mental Health** |  |  |  |  |  |  |  |  |  |  |
| K6 6-24 (severe psychological distress) % | 30.8 | 32.6 | 27.3 |  | 29.2 | 26.4 | 26.4 |  | 1.76 (0.63-4.96) | 0.78 (0.26-2.30) |
| K6 3-24 (medium-high psychological distress) % | 62.8 | 57.5 | 60.6 |  | 61.1 | 59.4 | 60.4 |  | 0.80 (0.30-2.11) | 0.73 (0.26-2.07) |
| *Restricted case analyses of K6* |  |  |  |  |  |  |  |  |  |  |
| ·         Analysis 1 K6 6-24^1^% | 21.8 | 23.8 | 22.4 |  | 19.5 | 20.8 | 17.6 |  | 1.01 (0.01-148.75) | 1.11 (0-279.23) |
| ·         Analysis 2 K6 6-24^2^ % | 24.8 | 28.2 | 26.6 |  | 22.5 | 24.4 | 20.8 |  | 1.02 (0.01-144.6) | 1.13 (0.00-270.69) |
| ·         Analysis 1 K6 3-24^1^ % | 49.2 | 51.8 | 55.2 |  | 49.6 | 51.9 | 51.7 |  | 1.00 (0.05-20.65) | 1.10 (0.04-27.89) |
| ·         Analysis 2 K6 3-24^2^ % | 54.8 | 60.6 | 65.5 |  | 56.6 | 61.8 | 60.3 |  | 1.14 (0.03-41.49) | 1.41 (0.03-65.05) |

Legend: * p<0.05; ** p<0.01; *** p<0.001

^1^ The outcomes of teachers that scored 6-24 (or 3-24) on the K6 at assessment 1.

^2^ The outcomes of teachers that scored 6-24 (or 3-24) on the K6 at assessment 1, but also excludes teachers that crossed over into the 6-24 (or 3-24) range at assessment 2 and/or 3.
