# Supplementary material for: Mental health first aid training for high school teachers: a cluster randomized trial
Source: BMC Psychiatry. 2010 Jun 24;10:51. doi: 10.1186/1471-244X-10-51 (PMC2908569; doi:10.1186/1471-244X-10-51)
Supplement: Additional file 2 — More detailed analyses of student outcome variables. [file 1471-244X-10-51-S2.DOCX]

### Additional file 2 – More detailed analyses of student outcome variables

**Table 1: Analyses of student outcome variables – 14 randomised schools**

|  | **Intervention group** | |  | **Control group** | |  | **Mean diff./OR for pre vs follow-up by intervention interaction (95% CI)** |
| --- | --- | --- | --- | --- | --- | --- | --- |
|  | **Pre** | **Follow-up** |  | **Pre** | **Follow-up** |  |  |
| **Mental Health Knowledge** |  |  |  |  |  |  |  |
| Recognition of depression *%* | 56.4 | 68.1 |  | 58.5 | 70.5 |  | 1.03 (0.67-1.59) |
| **Beliefs and Intentions About Where to Seek Help for Depression** |  |  |  |  |  |  |  |
| Help-seeking intentions - any adult source from 11 bullet point items below: *mean (SD)* | 3.79 (2.76) | 3.77 (2.91) |  | 3.67 (2.61) | 3.61 (2.81) |  | 0.01 (-0.30-0.32) |
| *Separate items for help-seeking intentions: % yes* |  |  |  |  |  |  |  |
| ·         Close family member | 81.7 | 78.2 |  | 83.8 | 80.7 |  | 1.01 (0.59-1.75) |
| ·         Teacher | 24.4 | 25.5 |  | 24.9 | 26.0 |  | 0.98 (0.65-1.50) |
| ·         School/student counsellor | 47.8 | 46.8 |  | 39.5 | 40.8 |  | 0.86 (0.58-1.29) |
| ·         Community member | 11.4 | 14.2 |  | 11.0 | 11.8 |  | 1.24 (0.71-2.14) |
| ·         Pastoral care worker | 18.6 | 18.8 |  | 17.7 | 20.5 |  | 0.79 (0.49-1.26) |
| ·         Community based religious leader | 13.4 | 13.7 |  | 12.0 | 13.4 |  | 0.83 (0.48-1.43) |
| ·         Telephone helpline or counselling service | 28.1 | 27.1 |  | 24.0 | 22.8 |  | 0.98 (0.62-1.55) |
| ·         GP or family doctor | 47.9 | 48.9 |  | 50.5 | 48.7 |  | 1.21 (0.82-1.79) |
| ·         CAMHS | 35.5 | 34.4 |  | 34.7 | 32.9 |  | 1.06 (0.71-1.57) |
| ·         Other mental health professionals | 33.4 | 34.7 |  | 34.3 | 33.3 |  | 1.16 (0.80-1.70) |
| ·         Youth health service | 37.5 | 35.5 |  | 34.1 | 32.2 |  | 0.98 (0.68-1.43) |
| Help-seeking intentions - all 11 adult source bullet point items above: *% yes* | 2.2 | 2.8 |  | 2.2 | 3.0 |  | 0.90 (0.31-2.58) |
| Help-seeking intentions - at least one adult source from the 11 bullet point items above: *% yes* | 92.3 | 90.4 |  | 93.6 | 91.2 |  | 1.18 (0.57-2.44) |
| Help-seeking intentions - all 5 items of those 11 bullet point items above^1^: *% yes* | 9.3 | 10.1 |  | 7.2 | 8.2 |  | 0.91 (0.49-1.70) |
| *Help-seeking beliefs: % helpful* |  |  |  |  |  |  |  |
| ·         School/student counsellor | 71.6 | 71.4 |  | 68.4 | 68.6 |  | 0.96 (0.63-1.44) |
| ·         Telephone helpline or counselling service | 52.0 | 49.0 |  | 47.3 | 43.2 |  | 1.05 (0.71-1.54) |
| ·         GP or family doctor | 65.8 | 64.3 |  | 65.4 | 64.9 |  | 0.93 (0.63-1.39) |
| ·         CAMHS | 59.0 | 59.7 |  | 59.1 | 57.2 |  | 1.18 (0.81-1.70) |
| ·         Other mental health professionals | 53.9 | 56.3 |  | 53.5 | 57.4 |  | 0.93 (0.65-1.32) |
| Help-seeking beliefs (all 5 bullet point items above): *% helpful* | 23.9 | 24.0 |  | 20.4 | 20.5 |  | 0.96 (0.61-1.52) |
| **Personal Stigma: % Strongly Disagree** |  |  |  |  |  |  |  |
| Could snap out of it | 12.5 | 16.5 |  | 13.9 | 19.9 |  | 0.84 (0.51-1.40) |
| Personal weakness | 12.3 | 14.6 |  | 15.5 | 19.5 |  | 0.89 (0.51-1.56) |
| Not real illness | 15.4 | 17.6 |  | 17.8 | 20.7 |  | 0.96 (0.60-1.55) |
| People with that problem are dangerous | 12.9 | 12.8 |  | 16.4 | 13.9 |  | 1.25 (0.76-2.06) |
| Best to avoid people with that problem | 34.7 | 33.6 |  | 36.4 | 38.1 |  | 0.85 (0.58-1.25) |
| People with that problem are unpredictable | 3.9 | 3.5 |  | 3.1 | 4.3 |  | 0.59 (0.25-1.41) |
| If they had problem they would not tell anyone | 21.9 | 19.8 |  | 27.4 | 22.7 |  | 1.26 (0.81-1.96) |
| *Alternative coding of the personal stigma items: % ≥ disagree* |  |  |  |  |  |  |  |
| ·         Could snap out of it | 50.7 | 57.4 |  | 58.4 | 61.2 |  | 1.32 (0.89-1.95) |
| ·         Personal weakness | 41.3 | 49.0 |  | 46.9 | 52.4 |  | 1.25 (0.84-1.86) |
| ·         Not real illness | 48.2 | 53.4 |  | 51.0 | 58.6 |  | 0.93 (0.65-1.35) |
| ·         People with that problem are dangerous | 46.8 | 48.2 |  | 48.6 | 53.0 |  | 0.84 (0.59-1.19) |
| ·         Best to avoid people with that problem | 74.6 | 76.2 |  | 78.1 | 78.6 |  | 1.14 (0.74-1.76) |
| ·         People with that problem are unpredictable | 20.4 | 19.5 |  | 16.6 | 20.0 |  | 0.71 (0.46-1.09) |
| ·         If they had problem they would not tell anyone | 62.1 | 57.9 |  | 64.6 | 59.9 |  | 1.15 (0.78-1.70) |
| **Perceived Stigma: % ≥ Agree** |  |  |  |  |  |  |  |
| Other people think could snap out of it | 47.9 | 46.0 |  | 43.5 | 41.3 |  | 1.00 (0.71-1.42) |
| Other people believe a sign of personal weakness | 52.2 | 53.0 |  | 52.5 | 46.9 |  | 1.42 (0.99-2.04) |
| Other people believe not real illness | 43.1 | 41.4 |  | 46.2 | 38.6 |  | 1.33 (0.95-1.86) |
| Other people believe they are dangerous | 37.4 | 38.2 |  | 39.0 | 34.4 |  | 1.34 (0.94-1.90) |
| Other people would avoid people with that problem | 37.4 | 38.4 |  | 39.0 | 37.7 |  | 1.13 (0.79-1.61) |
| Other people believe they are unpredictable | 44.1 | 47.6 |  | 53.7 | 48.2 |  | 1.64 (1.15-2.33)** |
| Other people would not tell anyone | 48.0 | 47.6 |  | 48.4 | 46.0 |  | 1.07 (0.76-1.51) |
| **Help Received from Teacher** |  |  |  |  |  |  |  |
| Talked with staff member about mental health problem: *% ≥ occasionally* | 5.2 | 6.7 |  | 2.4 | 4.2 |  | 0.67 (0.28-1.62) |
| *If "≥ occasionally" to item above, responses to the 6 bullet point items below* | |  |  |  |  |  |  |
| ·         Spent time listening: *% ≥ occasionally* | 37.3 | 47.5 |  | 24.2 | 33.3 |  | 0.56 (0.10-3.13) |
| ·         Helped calm down: *% ≥ occasionally* | 31.9 | 47.4 |  | 22.5 | 29.8 |  | 1.48 (0.58-3.78) |
| ·         Talked about suicidal thoughts: *% ≥ occasionally* | 12.8 | 24.0 |  | 7.4 | 7.7 |  | 2.95 (0.71-12.21) |
| ·         Recommended seeking professional help: *% ≥ occasionally* | 12.0 | 17.3 |  | 7.5 | 10.1 |  | 1.14 (0.35-3.72) |
| ·         Anything else:  *% ≥ occasionally* | 6.6 | 17.4 |  | 8.1 | 7.3 |  | 3.53 (0.72-17.28) |
| ·         How helpful: *% ≥ very helpful* | 36.2 | 41.1 |  | 29.0 | 30.0 |  | 1.28 (0.46-3.60) |
| Received information about mental health problems: *% yes* | 19.0 | 25.2 |  | 19.7 | 13.0 |  | 2.60 (1.68-4.05)*** |
| *If "yes" to item above, responses to the 5 bullet point items below: % yes* | |  |  |  |  |  |  |
| ·         Class lesson from teacher | 44.1 | 53.1 |  | 58.7 | 52.6 |  | 2.76 (1.10-6.91)* |
| ·         Poster, pamphlet, brochure or book | 32.5 | 51.9 |  | 47.7 | 32.6 |  | 4.84 (1.73-13.53)** |
| ·         Referral to website | 22.2 | 30.1 |  | 29.8 | 21.4 |  | 2.78 (1.02-7.55)* |
| ·         Talk from another person other than teacher | 28.6 | 45.4 |  | 30.1 | 37.9 |  | 1.53 (0.71-3.27) |
| ·         Something else | 19.6 | 25.2 |  | 27.4 | 21.9 |  | 3.54 (0.69-18.16) |
| **Student Mental Health** |  |  |  |  |  |  |  |
| SDQ 20-40 (abnormal) % | 9.1 | 9.6 |  | 7.0 | 10.3 |  | 0.51 (0.25-1.05) |
| SDQ 16-40 (borderline-abnormal) % | 21.9 | 21.1 |  | 16.8 | 19.9 |  | 0.58 (0.33-1.01) |
| *Restricted case analyses of SDQ* |  |  |  |  |  |  |  |
| ·         Analysis 1 SDQ 20-40 (abnormal)^2^ % | 9.1 | 8.5 |  | 7.0 | 5.9 |  | 1.05 (0.03-32.60) |
| ·         Analysis 2 SDQ 20-40 (abnormal)^3^ % | 9.3 | 8.7 |  | 7.2 | 6.1 |  | 1.06 (0.03-32.58) |
| ·         Analysis 1 SDQ 16-40 (borderline-abnormal)^2^ % | 21.9 | 20.3 |  | 16.8 | 15.1 |  | 0.99 (0.12-8.04) |
| ·         Analysis 2 SDQ 16-40 (borderline-abnormal)^3^ % | 23.3 | 22.3 |  | 18.2 | 16.7 |  | 1.00 (0.12-8.07) |
| *SDQ Subscales* |  |  |  |  |  |  |  |
| Emotional symptoms 7-10 (abnormal) % | 9.4 | 9.2 |  | 8.1 | 8.5 |  | 0.84 (0.42-1.70) |
| ·         Emotional symptoms 6-10 (borderline-abnormal) % | 15.1 | 14.1 |  | 14.3 | 13.1 |  | 0.99 (0.53-1.85) |
| Conduct problems 5-10 (abnormal) % | 9.6 | 9.0 |  | 7.8 | 9.2 |  | 0.68 (0.35-1.32) |
| ·         Conduct problems 4-10 (borderline-abnormal) % | 17.4 | 19.4 |  | 14.9 | 15.8 |  | 1.10 (0.63-1.93) |
| Hyperactivity 7-10 (abnormal) % | 16.2 | 16.2 |  | 14.7 | 15.8 |  | 0.90 (0.52-1.57) |
| ·         Hyperactivity 6-10 (borderline-abnormal) % | 27.9 | 25.8 |  | 23.5 | 22.3 |  | 0.97 (0.60-1.57) |
| Peer problems 6-10 (abnormal) % | 4.5 | 4.1 |  | 3.7 | 4.6 |  | 0.55 (0.21-1.45) |
| ·         Peer problems 4-10 (borderline-abnormal) % | 16.8 | 18.2 |  | 14.3 | 17.3 |  | 0.80 (0.48-1.33) |
| Prosocial behaviour 0-4 (abnormal) % | 10.8 | 10.5 |  | 10.3 | 9.0 |  | 1.09 (0.59-2.02) |
| ·         Prosocial behaviour 0-5 (borderline-abnormal) % | 21.0 | 22.0 |  | 23.1 | 21.8 |  | 1.15 (0.71-1.87) |

Legend: * p<0.05; ** p<0.01; *** p<0.001

^1^ The five intention items included nominating: a school/student counsellor, telephone helpline or counselling service, general practitioner or family doctor, child and adolescent mental health service (CAMHS), and other mental health professionals.

^2^ The outcomes of students that scored 20-40 (or 16-40) on the SDQ at assessment 1.

^3^ The outcomes of students that scored 20-40 (or 16-40) on the SDQ at assessment 1, but also excludes students that crossed over into the 20-40 (or 16-40) range at assessment 2.

**Table 2: Analyses of student outcome variables – 16 schools**

|  | **Intervention group** | |  | **Control group** | |  | **Mean diff./OR for pre vs follow-up by intervention interaction (95% CI)** |
| --- | --- | --- | --- | --- | --- | --- | --- |
|  | **Pre** | **Follow-up** |  | **Pre** | **Follow-up** |  |  |
| Recognition of depression *%* | 55.4 | 66.7 |  | 56.7 | 68.7 |  | 1.05 (0.69-1.61) |
| **Beliefs and Intentions About Where to Seek Help for Depression** |  |  |  |  |  |  |  |
| Help-seeking intentions - any adult source from 11 bullet point items below: *mean (SD)* | 3.80 (2.77) | 3.74 (2.91) |  | 3.74 (2.60) | 3.62 (2.81) |  | -0.03 (-0.32-0.27) |
| *Separate items for help-seeking intentions: % yes* |  |  |  |  |  |  |  |
| ·         Close family member | 81.3 | 77.4 |  | 84.5 | 80.6 |  | 1.06 (0.62-1.80) |
| ·         Teacher | 25.1 | 25.1 |  | 25.4 | 26.5 |  | 0.90 (0.60-1.35) |
| ·         School/student counsellor | 46.9 | 45.9 |  | 39.8 | 40.4 |  | 0.90 (0.61-1.33) |
| ·         Community member | 11.7 | 13.9 |  | 10.9 | 11.4 |  | 1.16 (0.68-1.97) |
| ·         Pastoral care worker | 18.1 | 18.1 |  | 17.2 | 20.6 |  | 0.74 (0.47-1.17) |
| ·         Community based religious leader | 13.7 | 13.8 |  | 11.6 | 13.5 |  | 0.76 (0.44-1.31) |
| ·         Telephone helpline or counselling service | 29.1 | 27.7 |  | 25.4 | 23.3 |  | 0.97 (0.63-1.49) |
| ·         GP or family doctor | 48.6 | 49.0 |  | 50.9 | 49.6 |  | 1.11 (0.76-1.61) |
| ·         CAMHS | 35.4 | 33.7 |  | 36.3 | 32.4 |  | 1.15 (0.78-1.67) |
| ·         Other mental health professionals | 33.7 | 34.5 |  | 35.4 | 32.9 |  | 1.20 (0.83-1.73) |
| ·         Youth health service | 38.2 | 34.6 |  | 35.7 | 32.8 |  | 0.92 (0.64-1.33) |
| Help-seeking intentions - all 11 adult source bullet point items above: *% yes* | 2.2 | 2.8 |  | 2.0 | 2.8 |  | 0.84 (0.30-2.36) |
| Help-seeking intentions - at least one adult source from the 11 bullet point items above: *% yes* | 92.1 | 90.0 |  | 93.8 | 91.1 |  | 1.19 (0.59-2.41) |
| Help-seeking intentions - all 5 items of those 11 bullet point items above^1^: *% yes* | 9.4 | 10.7 |  | 7.3 | 7.8 |  | 1.05 (0.57-1.91) |
| *Help-seeking beliefs: % helpful* |  |  |  |  |  |  |  |
| ·         School/student counsellor | 69.7 | 69.4 |  | 67.3 | 68.0 |  | 0.96 (0.65-1.42) |
| ·         Telephone helpline or counselling service | 52.2 | 48.0 |  | 47.9 | 44.3 |  | 0.92 (0.64-1.33) |
| ·         GP or family doctor | 65.3 | 63.8 |  | 65.7 | 65.5 |  | 0.90 (0.61-1.32) |
| ·         CAMHS | 58.6 | 58.3 |  | 59.9 | 56.8 |  | 1.19 (0.83-1.70) |
| ·         Other mental health professionals | 53.0 | 54.9 |  | 53.6 | 56.6 |  | 0.95 (0.67-1.33) |
| Help-seeking beliefs (all 5 bullet point items above): *% helpful* | 23.4 | 23.0 |  | 20.7 | 20.2 |  | 0.96 (0.62-1.49) |
| **Personal Stigma: % Strongly Disagree** |  |  |  |  |  |  |  |
| Could snap out of it | 12.9 | 16.3 |  | 13.6 | 19.7 |  | 0.77 (0.47-1.25) |
| Personal weakness | 12.0 | 14.5 |  | 15.0 | 19.4 |  | 0.92 (0.53-1.57) |
| Not real illness | 14.7 | 16.9 |  | 17.9 | 20.6 |  | 1.00 (0.63-1.58) |
| People with that problem are dangerous | 13.3 | 12.8 |  | 16.2 | 14.6 |  | 1.09 (0.68-1.75) |
| Best to avoid people with that problem | 33.7 | 32.9 |  | 35.6 | 38.4 |  | 0.83 (0.57-1.21) |
| People with that problem are unpredictable | 3.8 | 3.5 |  | 3.1 | 4.8 |  | 0.54 (0.24-1.23) |
| If they had problem they would not tell anyone | 21.8 | 19.9 |  | 26.8 | 23.5 |  | 1.17 (0.77-1.78) |
| *Alternative coding of the personal stigma items: % ≥ disagree* |  |  |  |  |  |  |  |
| ·         Could snap out of it | 49.9 | 56.6 |  | 57.2 | 61.5 |  | 1.23 (0.85-1.79) |
| ·         Personal weakness | 40.4 | 47.3 |  | 45.5 | 52.4 |  | 1.14 (0.78-1.68) |
| ·         Not real illness | 46.5 | 51.8 |  | 50.3 | 58.7 |  | 0.91 (0.64-1.30) |
| ·         People with that problem are dangerous | 47.4 | 49.0 |  | 48.7 | 53.8 |  | 0.81 (0.58-1.14) |
| ·         Best to avoid people with that problem | 73.1 | 74.9 |  | 77.1 | 79.1 |  | 1.08 (0.72-1.63) |
| ·         People with that problem are unpredictable | 19.8 | 19.8 |  | 17.5 | 20.7 |  | 0.77 (0.51-1.16) |
| ·         If they had problem they would not tell anyone | 61.9 | 58.3 |  | 63.7 | 60.0 |  | 1.14 (0.79-1.66) |
| **Perceived Stigma: % ≥ Agree** |  |  |  |  |  |  |  |
| Other people think could snap out of it | 48.4 | 45.8 |  | 44.8 | 41.6 |  | 1.01 (0.72-1.42) |
| Other people believe a sign of personal weakness | 52.9 | 53.3 |  | 53.0 | 47.5 |  | 1.37 (0.96-1.93) |
| Other people believe not real illness | 43.4 | 41.4 |  | 45.7 | 39.0 |  | 1.27 (0.92-1.75) |
| Other people believe they are dangerous | 37.1 | 38.5 |  | 40.5 | 35.2 |  | 1.41 (1.01-1.98)* |
| Other people would avoid people with that problem | 37.7 | 38.3 |  | 39.6 | 38.0 |  | 1.11 (0.79-1.57) |
| Other people believe they are unpredictable | 44.4 | 46.1 |  | 54.1 | 48.9 |  | 1.46 (1.04-2.04)* |
| Other people would not tell anyone | 47.9 | 47.3 |  | 50.2 | 46.1 |  | 1.14 (0.82-1.58) |
| **Help Received from Teacher** |  |  |  |  |  |  |  |
| Talked with staff member about mental health problem: *% ≥ occasionally* | 5.4 | 6.5 |  | 3.0 | 4.9 |  | 0.63 (0.28-1.42) |
| *If "≥ occasionally" to item above, responses to the 6 bullet point items below* |  |  |  |  |  |  |  |
| ·         Spent time listening: *% ≥ occasionally* | 35.6 | 44.2 |  | 25.3 | 35.6 |  | 0.68 (0.21-2.22) |
| ·         Helped calm down: *% ≥ occasionally* | 31.9 | 42.8 |  | 24.3 | 29.4 |  | 1.28 (0.56-2.95) |
| ·         Talked about suicidal thoughts: *% ≥ occasionally* | 13.0 | 23.4 |  | 7.1 | 7.1 |  | 2.61 (0.72-9.43) |
| ·         Recommended seeking professional help: *% ≥ occasionally* | 11.6 | 17.2 |  | 7.9 | 11.3 |  | 1.07 (0.38-3.03) |
| ·         Anything else:  *% ≥ occasionally* | 5.9 | 15.5 |  | 8.4 | 6.7 |  | 4.17 (0.88-19.75) |
| ·         How helpful: *% ≥ very helpful* | 34.8 | 39.7 |  | 28.2 | 28.6 |  | 1.26 (0.49-3.23) |
| Received information about mental health problems: *% yes* | 20.0 | 24.2 |  | 20.3 | 13.4 |  | 2.20 (1.45-3.34)*** |
| *If "yes" to item above, responses to the 5 bullet point items below: % yes* |  |  |  |  |  |  |  |
| ·         Class lesson from teacher | 47.9 | 53.0 |  | 59.6 | 52.9 |  | 2.36 (0.99-5.61) |
| ·         Poster, pamphlet, brochure or book | 32.7 | 48.9 |  | 47.7 | 32.3 |  | 3.96 (1.63-9.63)** |
| ·         Referral to website | 23.0 | 30.1 |  | 26.6 | 19.8 |  | 2.67 (1.01-7.09)* |
| ·         Talk from another person other than teacher | 31.3 | 42.7 |  | 33.3 | 37.2 |  | 1.43 (0.70-2.93) |
| ·         Something else | 17.5 | 26.0 |  | 24.5 | 20.3 |  | 3.68 (0.93-14.48) |
| **Student Mental Health** |  |  |  |  |  |  |  |
| SDQ 20-40 (abnormal) % | 9.8 | 10.2 |  | 8.2 | 10.5 |  | 0.56 (0.28-1.11) |
| SDQ 16-40 (borderline-abnormal) % | 22.4 | 22.2 |  | 18.8 | 20.4 |  | 0.65 (0.38-1.11) |
| *Restricted case analyses of SDQ* |  |  |  |  |  |  |  |
| ·         Analysis 1 SDQ 20-40 (abnormal)^2^ % | 9.8 | 8.9 |  | 8.2 | 6.3 |  | 1.17 (0.05-26.84) |
| ·         Analysis 2 SDQ 20-40 (abnormal)^3^ % | 9.9 | 9.2 |  | 8.4 | 6.6 |  | 1.18 (0.05-26.80) |
| ·         Analysis 1 SDQ 16-40 (borderline-abnormal)^2^ % | 22.4 | 20.7 |  | 18.8 | 15.3 |  | 1.14 (0.16-8.10) |
| ·         Analysis 2 SDQ 16-40 (borderline-abnormal)^3^ % | 24.0 | 23.0 |  | 20.3 | 16.9 |  | 1.15 (0.16-8.11) |
| *SDQ Subscales* |  |  |  |  |  |  |  |
| Emotional symptoms 7-10 (abnormal) % | 9.5 | 9.7 |  | 8.5 | 8.8 |  | 0.92 (0.46-1.82) |
| ·         Emotional symptoms 6-10 (borderline-abnormal) % | 15.4 | 14.8 |  | 14.5 | 13.3 |  | 1.03 (0.56-1.87) |
| Conduct problems 5-10 (abnormal) % | 9.9 | 9.4 |  | 9.4 | 9.3 |  | 0.81 (0.42-1.54) |
| ·         Conduct problems 4-10 (borderline-abnormal) % | 17.6 | 19.6 |  | 17.0 | 16.3 |  | 1.14 (0.66-1.95) |
| Hyperactivity 7-10 (abnormal) % | 16.1 | 16.9 |  | 15.9 | 16.1 |  | 1.01 (0.59-1.72) |
| ·         Hyperactivity 6-10 (borderline-abnormal) % | 28.1 | 26.4 |  | 25.6 | 22.8 |  | 1.05 (0.66-1.68) |
| Peer problems 6-10 (abnormal) % | 4.7 | 4.7 |  | 4.0 | 4.6 |  | 0.72 (0.29-1.74) |
| ·         Peer problems 4-10 (borderline-abnormal) % | 17.9 | 19.9 |  | 14.9 | 17.9 |  | 0.81 (0.50-1.31) |
| Prosocial behaviour 0-4 (abnormal) % | 11.0 | 10.6 |  | 9.9 | 8.9 |  | 1.04 (0.57-1.91) |
| ·         Prosocial behaviour 0-5 (borderline-abnormal) % | 22.0 | 22.7 |  | 22.5 | 21.1 |  | 1.12 (0.70-1.78) |

Legend: * p<0.05; ** p<0.01; *** p<0.001

^1^ The five intention items included nominating: a school/student counsellor, telephone helpline or counselling service, general practitioner or family doctor, child and adolescent mental health service (CAMHS), and other mental health professionals.

^2^ The outcomes of students that scored 20-40 (or 16-40) on the SDQ at assessment 1.

^3^ The outcomes of students that scored 20-40 (or 16-40) on the SDQ at assessment 1, but also excludes students that crossed over into the 20-40 (or 16-40) range at assessment 2.
